# Different types of quantum integral inequalities via $(\alpha ,m)$-convexity

**DOI:** 10.1186/s13660-018-1860-2

**Published:** 2018-09-27

**Authors:** Yao Zhang, Ting-Song Du, Hao Wang, Yan-Jun Shen

**Affiliations:** 1 Department of Mathematics, College of Science, China Three Gorges University, Yichang, China; 2 Three Gorges Mathematical Research Center, China Three Gorges University, Yichang, China; 3 Hubei Provincial Collaborative Innovation Center for New Energy Microgrid, China Three Gorges University, Yichang, China

**Keywords:** 34A08, 26A51, 26D10, 26D15, Quantum integral inequalities, $(\alpha, m)$-convex functions, Hermite–Hadamard’s inequality, Simpson’s inequality

## Abstract

In this paper, based on $(\alpha,m)$-convexity, we establish different type inequalities via quantum integrals. These inequalities generalize some results given in the literature.

## Introduction and preliminaries

Throughout the paper, let $I:= [a, b]\subseteq\mathbb{R}$ with $0\leq a< b$ be an interval, $I^{\circ}$ be the interior of *I* and let $0< q<1$ be a constant.

Let $f:I\rightarrow\mathbb{R}$ be convex on *I*, then the Hermite–Hadamard inequality holds:

1.1 $$ f \biggl(\frac{a+b}{2} \biggr)\leq\frac{1}{b-a} \int^{b}_{a} f(x)\, \mathrm{d}x\leq \frac{f(a)+f(b)}{2}. $$

If $f:I\rightarrow\mathbb{R}$ is four times continuously differentiable on $I^{\circ}$ and $\|f^{(4)} \|_{\infty}= \sup_{x\in (a,b)} |f^{(4)}(x) |<\infty$, then the Simpson inequality holds:

1.2 $$ \biggl\vert \frac{1}{3}\biggl[\frac{f(a)+f(b)}{2}+2f \biggl(\frac{a+b}{2} \biggr)\biggr]-\frac{1}{b-a} \int^{b}_{a} f(x)\, \mathrm{d}x \biggr\vert \leq \frac {1}{2880} \bigl\Vert f^{(4)} \bigr\Vert _{\infty}(b-a)^{4}. $$

Many researchers generalized the inequalities (1.1) and (1.2). For more details on these inequalities, see [5–8, 10–14, 16, 17, 22, 24, 25].

In 2014, Tariboon and Ntouyas defined the *q*-derivative and *q*-integral as follows.

### Definition 1.1

([28])

Let $f: I\rightarrow\mathbb{R}$ be a continuous function and let $x\in I$. Then the *q*-derivative on *I* of *f* at *x* is defined as

$$ {}_{a}D_{q}f(x)=\frac{f(x)-f(qx+(1-q)a)}{(1-q)(x-a)}, \quad x\neq a,\qquad {}_{a}D_{q}f(a)={\lim_{x\to a}} {}_{a}D_{q}f(x). $$

### Definition 1.2

([28])

Let $f: I\rightarrow\mathbb{R}$ be a continuous function. Then the *q*-integral on *I* is defined as

$$ \int^{x}_{a}f(t)\, {}_{a} \mathrm{d}_{q}t=(1-q) (x-a)\sum^{\infty}_{n=0}q^{n}f \bigl(q^{n}x+\bigl(1-q^{n}\bigr)a \bigr) $$

for $x\in I$. Moreover, if $c\in(a,x)$, then the *q*-integral on *I* is defined as

$$ \int^{x}_{c}f(t)\, {}_{a} \mathrm{d}_{q}t= \int^{x}_{a}f(t)\, {}_{a} \mathrm{d}_{q}t- \int ^{c}_{a}f(t)\, {}_{a} \mathrm{d}_{q}t. $$

In the same paper, they also proved the following *q*-Hölder inequality.

### Theorem 1.1

([28])

*Let* $f,g: I\rightarrow\mathbb{R}$ *be two continuous functions*. *Then the inequality*

$$ \int^{x}_{a} \bigl\vert f(t) \bigr\vert \bigl\vert g(t) \bigr\vert \, {}_{a}\mathrm{d}_{q}t\leq \biggl( \int ^{x}_{a} \bigl\vert f(t) \bigr\vert ^{r_{1}}\, {}_{a}\mathrm{d}_{q}t \biggr)^{\frac{1}{r_{1}}} \biggl( \int^{x}_{a} \bigl\vert g(t) \bigr\vert ^{r_{2}}\, {}_{a}\mathrm{d}_{q}t \biggr)^{\frac{1}{r_{2}}} $$

*holds for all* $x\in I$ *and* $r_{1},r_{2}>1$ *with* $r_{1}^{-1}+r_{2}^{-1}=1$.

In 2018, Alp et al. generalized the Hermite–Hadamard inequality to the form of *q*-integrals as follows.

### Theorem 1.2

([2])

*Let* $f:I\rightarrow\mathbb{R}$ *be convex and differentiable on* *I* *with* $0< q<1$. *Then we have*

1.3 $$ f \biggl(\frac{qa+b}{1+q} \biggr)\leq\frac{1}{b-a} \int_{a}^{b}f(x)\, {}_{a}\mathrm {d}_{q}x\leq\frac{qf(a)+f(b)}{1+q}. $$

For more details on the inequality (1.3), see [15, 18, 20, 21, 23]. For other type quantum integral inequalities, the interested reader can refer to [3, 4, 27, 29, 31].

In 1993, Miheşan gave the definition of $(\alpha,m)$-convex functions as follows.

### Definition 1.3

([19])

For $b^{*}>0$, the function $f: [0,b^{*}]\rightarrow\mathbb{R}$ is named $(\alpha,m)$-convex with $\alpha,m\in(0,1]$ if the inequality

$$ f \bigl(tx+m(1-t)y \bigr)\leq t^{\alpha}f(x)+m\bigl(1-t^{\alpha} \bigr)f(y) $$

holds for all $x,y\in[0,b^{*}]$ and $t\in[0,1]$.

This paper aims to establish different types of quantum integral inequalities via $(\alpha,m)$-convexity. Some relevant connections of the results obtained in this paper with previous ones are also pointed out.

## Auxiliary results

For proving main results, we need the following lemma.

### Lemma 2.1

*Let* $f: I\rightarrow\mathbb{R}$ *be a continuous and* *q*-*differentiable function on* $I^{\circ}$ *with* $0< q <1$. *Then the identity*

$$\begin{aligned} &\lambda \bigl[\mu f(b)+(1-\mu)f(a) \bigr]+(1-\lambda)f \bigl(\mu b+(1-\mu )a \bigr)-\frac{1}{b-a} \int_{a}^{b}f(x)\,{}_{a} \mathrm{d}_{q}x \\ &\quad =(b-a) \biggl\{ \int_{0}^{\mu}(qt+\lambda\mu-\lambda){}_{a}D_{q}f \bigl(tb+(1-t)a \bigr)\,{}_{0}\mathrm{d}_{q}t \\ &\qquad {} + \int_{\mu}^{1} (qt+\lambda\mu-1){}_{a}D_{q}f \bigl(tb+(1-t)a \bigr)\,{}_{0}\mathrm {d}_{q}t \biggr\} \end{aligned}$$

*holds for all* $\lambda,\mu\in[0,1]$ *if* ${}_{a}D_{q}f$ *is integrable on* *I*.

### Proof

By an identical transformation, we get

2.1 $$\begin{aligned} &(b-a) \biggl\{ \int_{0}^{\mu}(qt+\lambda\mu-\lambda){}_{a}D_{q}f \bigl(tb+(1-t)a \bigr)\,{}_{0}\mathrm{d}_{q}t \\ &\qquad {} + \int_{\mu}^{1} (qt+\lambda\mu-1){}_{a}D_{q}f \bigl(tb+(1-t)a \bigr)\,{}_{0}\mathrm {d}_{q}t \biggr\} \\ &\quad =(b-a) \biggl\{ \int_{0}^{1} (qt+\lambda\mu-1){}_{a}D_{q}f \bigl(tb+(1-t)a \bigr)\,{}_{0}\mathrm{d}_{q}t \\ &\qquad {} + \int_{0}^{\mu}(1-\lambda) {}_{a}D_{q}f \bigl(tb+(1-t)a \bigr)\,{}_{0}\mathrm {d}_{q}t \biggr\} . \end{aligned}$$

From Definition 1.1, we get

$$\begin{aligned} {}_{a}D_{q}f \bigl(tb+(1-t)a \bigr)&=\frac {f(tb+(1-t)a)-f(q[tb+(1-t)a]+(1-q)a)}{(1-q)(tb+(1-t)a-a)} \\ &=\frac{f(tb+(1-t)a)-f(qtb+(1-qt)a)}{t(1-q)(b-a)}. \end{aligned}$$

Utilizing the above calculation and Definition 1.2, we have

2.2 $$\begin{aligned} & \int_{0}^{1}t {}_{a}D_{q}f \bigl(tb+(1-t)a \bigr)\,{}_{0}\mathrm{d}_{q}t \\ &\quad = \int_{0}^{1} \frac{f(tb+(1-t)a)-f(qtb+(1-qt)a)}{(1-q)(b-a)}\,{}_{0} \mathrm {d}_{q}t \\ &\quad =\frac{1}{b-a} \Biggl\{ \sum_{n=0}^{\infty}q^{n} f \bigl(q^{n} b+\bigl(1-q^{n}\bigr)a \bigr) \\ &\qquad {} -\sum_{n=0}^{\infty}q^{n} f \bigl(q^{n+1} b+\bigl(1-q^{n+1}\bigr)a \bigr) \Biggr\} \\ &\quad =\frac{1}{b-a} \Biggl\{ \sum_{n=0}^{\infty}q^{n} f \bigl(q^{n} b+\bigl(1-q^{n}\bigr)a \bigr) \\ &\qquad {} -\frac{1}{q}\sum_{n=0}^{\infty}q^{n+1} f \bigl(q^{n+1} b+\bigl(1-q^{n+1}\bigr)a \bigr) \Biggr\} \\ &\quad =\frac{1}{b-a} \Biggl\{ f(b)+ \biggl(1-\frac{1}{q} \biggr)\sum _{n=1}^{\infty }q^{n} f \bigl(q^{n} b+\bigl(1-q^{n}\bigr)a \bigr) \Biggr\} \\ &\quad =\frac{1}{b-a} \Biggl\{ \frac{1}{q}f(b)-\frac{1-q}{q}\sum _{n=0}^{\infty }q^{n} f \bigl(q^{n} b+\bigl(1-q^{n}\bigr)a \bigr) \Biggr\} \\ &\quad =\frac{f(b)}{q(b-a)}-\frac{1}{q(b-a)^{2}} \int_{a}^{b}f(x)\,{}_{a} \mathrm{d}_{q}x, \end{aligned}$$

2.3 $$\begin{aligned} & \int_{0}^{1}{}_{a}D_{q}f \bigl(tb+(1-t)a \bigr)\,{}_{0}\mathrm{d}_{q}t \\ &\quad = \int_{0}^{1} \frac{f(tb+(1-t)a)-f(qtb+(1-qt)a)}{t(1-q)(b-a)}\,{}_{0} \mathrm {d}_{q}t \\ &\quad =\frac{1}{b-a} \Biggl\{ \sum_{n=0}^{\infty} f \bigl(q^{n} b+\bigl(1-q^{n}\bigr)a \bigr)-\sum _{n=0}^{\infty} f \bigl(q^{n+1} b+ \bigl(1-q^{n+1}\bigr)a \bigr) \Biggr\} \\ &\quad =\frac{f(b)-f(a)}{b-a} \end{aligned}$$

and

2.4 $$\begin{aligned} & \int_{0}^{\mu}{}_{a}D_{q}f \bigl(tb+(1-t)a \bigr)\,{}_{0}\mathrm{d}_{q}t \\ &\quad = \int_{0}^{\mu}\frac {f(tb+(1-t)a)-f(qtb+(1-qt)a)}{t(1-q)(b-a)}\,{}_{0} \mathrm{d}_{q}t \\ &\quad =\frac{1}{b-a} \Biggl\{ \sum_{n=0}^{\infty} f \bigl(q^{n}\mu b+\bigl(1-q^{n}\mu \bigr)a \bigr) -\sum _{n=0}^{\infty} f \bigl(q^{n+1}\mu b+ \bigl(1-q^{n+1}\mu\bigr)a \bigr) \Biggr\} \\ &\quad =\frac{f(\mu b+(1-\mu)a)-f(a)}{b-a}. \end{aligned}$$

Substituting (2.2), (2.3) and (2.4) into (2.1), we can obtain the desired result. This ends the proof. □

### Remark 2.1

In Lemma 2.1, if one takes $q\rightarrow1^{-}$, one has [9, Lemma 2].

### Remark 2.2

Consider Lemma 2.1.

(i) Putting $\mu=0$, we have
  2.5 $$\begin{aligned}& f(a)-\frac{1}{b-a} \int_{a}^{b}f(x)\,{}_{a} \mathrm{d}_{q}x \\& \quad =(b-a) \int_{0}^{1} (qt-1){}_{a}D_{q}f \bigl(tb+(1-t)a \bigr)\,{}_{0}\mathrm{d}_{q}t. \end{aligned}$$
(ii) Putting $\mu=1$, we have
  2.6 $$ f(b)-\frac{1}{b-a} \int_{a}^{b}f(x)\,{}_{a} \mathrm{d}_{q}x =(b-a) \int_{0}^{1} qt {}_{a}D_{q}f \bigl(tb+(1-t)a \bigr)\,{}_{0}\mathrm{d}_{q}t. $$
(iii) Putting $\mu=\frac{1}{1+q}$, we have
  2.7 $$\begin{aligned} &\lambda\frac{qf(a)+f(b)}{1+q}+(1-\lambda)f \biggl(\frac{qa+b}{1+q} \biggr)- \frac{1}{b-a} \int_{a}^{b}f(x)\,{}_{a} \mathrm{d}_{q}x \\ &\quad =(b-a) \biggl\{ \int_{0}^{\frac{1}{1+q}} \biggl(qt-\frac{\lambda q}{1+q} \biggr){}_{a}D_{q}f \bigl(tb+(1-t)a \bigr) \,{}_{0}\mathrm{d}_{q}t \\ &\qquad {} + \int_{\frac{1}{1+q}}^{1} \biggl(qt+\frac{\lambda}{1+q}-1 \biggr){}_{a}D_{q}f \bigl(tb+(1-t)a \bigr) \,{}_{0}\mathrm{d}_{q}t \biggr\} . \end{aligned}$$

### Remark 2.3

Consider Lemma 2.1.

(i) Putting $\lambda=0$, we get

2.8 $$\begin{aligned} &f \bigl(\mu b+(1-\mu)a \bigr)-\frac{1}{b-a} \int_{a}^{b}f(x)\,{}_{a} \mathrm{d}_{q}x \\ &\quad =(b-a) \biggl\{ \int_{0}^{\mu}qt {}_{a}D_{q}f \bigl(tb+(1-t)a \bigr)\,{}_{0}\mathrm {d}_{q}t \\ &\qquad {} + \int_{\mu}^{1} (qt-1){}_{a}D_{q}f \bigl(tb+(1-t)a \bigr)\,{}_{0}\mathrm{d}_{q}t \biggr\} . \end{aligned}$$

Specially, taking $\mu=\frac{1}{1+q}$, we obtain the midpoint-like integral identity

$$\begin{aligned} &f \biggl({\frac{qa+b}{1+q}} \biggr)-\frac{1}{b-a} \int_{a}^{b}f(x)\,{}_{a}\mathrm {d}_{q}x \\ &\quad =(b-a) \biggl\{ \int_{0}^{\frac{1}{1+q}} qt {}_{a}D_{q}f \bigl(tb+(1-t)a \bigr)\,{}_{0}\mathrm{d}_{q}t \\ &\qquad {} + \int_{\frac{1}{1+q}}^{1} (qt-1){}_{a}D_{q}f \bigl(tb+(1-t)a \bigr)\,{}_{0}\mathrm {d}_{q}t \biggr\} , \end{aligned}$$

which is presented by Alp et al. in [2, Lemma 11].

(ii) Putting $\lambda=\frac{1}{3}$, we get

2.9 $$\begin{aligned} &\frac{1}{3} \bigl[\mu f(b)+(1-\mu)f(a)+2f \bigl(\mu b+(1-\mu)a \bigr) \bigr]-\frac{1}{b-a} \int_{a}^{b}f(x)\,{}_{a} \mathrm{d}_{q}x \\ &\quad =(b-a) \biggl\{ \int_{0}^{\mu}\biggl(qt+\frac{1}{3}\mu- \frac{1}{3} \biggr){}_{a}D_{q}f \bigl(tb+(1-t)a \bigr)\,{}_{0}\mathrm{d}_{q}t \\ &\qquad {} + \int_{\mu}^{1} \biggl(qt+\frac{1}{3}\mu-1 \biggr){}_{a}D_{q}f \bigl(tb+(1-t)a \bigr) \,{}_{0}\mathrm{d}_{q}t \biggr\} . \end{aligned}$$

Specially, taking $\mu=\frac{1}{1+q}$, we obtain the Simpson-like integral identity

2.10 $$\begin{aligned} &\frac{1}{3} \biggl[\frac{qf(a)+f(b)}{1+q}+2f \biggl(\frac{qa+b}{1+q} \biggr) \biggr]-\frac{1}{b-a} \int_{a}^{b}f(x)\,{}_{a} \mathrm{d}_{q}x \\ &\quad =(b-a) \biggl\{ \int_{0}^{\frac{1}{1+q}} \biggl(qt-\frac{q}{3+3q} \biggr){}_{a}D_{q}f \bigl(tb+(1-t)a \bigr) \,{}_{0}\mathrm{d}_{q}t \\ &\qquad {} + \int_{\frac{1}{1+q}}^{1} \biggl(qt+\frac{1}{3+3q}-1 \biggr){}_{a}D_{q}f \bigl(tb+(1-t)a \bigr) \,{}_{0}\mathrm{d}_{q}t \biggr\} . \end{aligned}$$

(iii) Putting $\lambda=\frac{1}{2}$, we get

2.11 $$\begin{aligned} &\frac{1}{2} \bigl[\mu f(b)+(1-\mu)f(a)+f \bigl(\mu b+(1-\mu)a \bigr) \bigr]-\frac{1}{b-a} \int_{a}^{b}f(x)\,{}_{a} \mathrm{d}_{q}x \\ &\quad =(b-a) \biggl\{ \int_{0}^{\mu}\biggl(qt+\frac{1}{2}\mu- \frac{1}{2} \biggr){}_{a}D_{q}f \bigl(tb+(1-t)a \bigr)\,{}_{0}\mathrm{d}_{q}t \\ &\qquad {} + \int_{\mu}^{1} \biggl(qt+\frac{1}{2}\mu-1 \biggr){}_{a}D_{q}f \bigl(tb+(1-t)a \bigr) \,{}_{0}\mathrm{d}_{q}t \biggr\} . \end{aligned}$$

Specially, taking $\mu=\frac{1}{1+q}$, we obtain the averaged midpoint-trapezoid-like integral identity

2.12 $$\begin{aligned} &\frac{1}{2} \biggl[\frac{qf(a)+f(b)}{1+q}+f \biggl(\frac{qa+b}{1+q} \biggr) \biggr]-\frac{1}{b-a} \int_{a}^{b}f(x)\,{}_{a} \mathrm{d}_{q}x \\ &\quad =(b-a) \biggl\{ \int_{0}^{\frac{1}{1+q}} \biggl(qt-\frac{q}{2+2q} \biggr){}_{a}D_{q}f \bigl(tb+(1-t)a \bigr) \,{}_{0}\mathrm{d}_{q}t \\ &\qquad {} + \int_{\frac{1}{1+q}}^{1} \biggl(qt+\frac{1}{2+2q}-1 \biggr){}_{a}D_{q}f \bigl(tb+(1-t)a \bigr) \,{}_{0}\mathrm{d}_{q}t \biggr\} . \end{aligned}$$

(iv) Putting $\lambda=1$, we get

2.13 $$\begin{aligned} &\mu f(b)+(1-\mu)f(a)-\frac{1}{b-a} \int_{a}^{b}f(x)\,{}_{a} \mathrm{d}_{q}x \\ &\quad =(b-a) \int_{0}^{1} (qt+\mu-1){}_{a}D_{q}f \bigl(tb+(1-t)a \bigr)\,{}_{0}\mathrm{d}_{q}t. \end{aligned}$$

Specially, taking $\mu=\frac{1}{1+q}$, we obtain the trapezoid-like integral identity

$$\begin{aligned} &\frac{qf(a)+f(b)}{1+q}-\frac{1}{b-a} \int_{a}^{b}f(x)\,{}_{a} \mathrm{d}_{q}x \\ &\quad =(b-a) \int_{0}^{1} \biggl(qt+\frac{1}{1+q}-1 \biggr){}_{a}D_{q}f \bigl(tb+(1-t)a \bigr) \,{}_{0}\mathrm{d}_{q}t, \end{aligned}$$

which is presented by Sudsutad et al. in [26, Lemma 3.1].

It is worth to mention here that to the best of our knowledge the obtained identities (2.5)–(2.13) are new in the literature.

Next we provide some calculations which will be used in this paper.

### Lemma 2.2

*Let* $\mu\in[0,1]$ *and* $\tau\in[0,\infty)$. *From Definition *1.2, *we have*

$$ \int_{0}^{\mu}t^{\tau} \,{}_{0} \mathrm{d}_{q}t=(1-q)\sum_{n=0}^{\infty} \mu^{\tau +1}q^{(\tau+1)n} =\frac{\mu^{\tau+1}(1-q)}{1-q^{\tau+1}} $$

*and*

$$ \int_{0}^{\mu}(1-t)^{\tau} \,{}_{0}\mathrm{d}_{q}t=(1-q)\mu\sum _{n=0}^{\infty }q^{n} \bigl(1-q^{n}\mu \bigr)^{\tau}. $$

### Lemma 2.3

*Let* $\lambda,\mu\in[0,1]$ *and* $\tau\in[0,\infty)$. *Then we have*

$$\begin{aligned} & \int_{0}^{\mu}t^{\tau} \bigl\vert qt-( \lambda-\lambda\mu) \bigr\vert \,{}_{0}\mathrm {d}_{q}t \\ & \quad = \textstyle\begin{cases} \frac{\mu^{\tau+1}(1-q)(\lambda-\lambda\mu)}{1-q^{\tau+1}}-\frac{q\mu ^{\tau+2}(1-q)}{1-q^{\tau+2}}, &(\lambda+q)\mu\leq\lambda, \\ \frac{2(1-q)^{2}(\lambda-\lambda\mu)^{\tau+2}}{(1-q^{\tau+1})(1-q^{\tau +2})}+\frac{q\mu^{\tau+2}(1-q)}{1-q^{\tau+2}}-\frac{\mu^{\tau +1}(1-q)(\lambda-\lambda\mu)}{1-q^{\tau+1}}, &(\lambda+q)\mu>\lambda, \end{cases}\displaystyle \end{aligned}$$

*and*

$$\begin{aligned} & \int_{0}^{\mu}(1-t)^{\tau} \bigl\vert qt-( \lambda-\lambda\mu) \bigr\vert \,{}_{0}\mathrm {d}_{q}t \\ &\quad = \textstyle\begin{cases} (1-q)\mu\sum_{n=0}^{\infty}q^{n} (\lambda-\lambda\mu-q^{n+1}\mu ) (1-q^{n}\mu )^{\tau}, &(\lambda+q)\mu\leq\lambda, \\ \left [ \textstyle\begin{array}{l} 2(1-q)(\lambda-\lambda\mu)^{2}\sum_{n=0}^{\infty}q^{n-1} (1-q^{n} ) [1-q^{n-1}(\lambda-\lambda\mu) ]^{\tau} \\ \quad {}-(1-q)\mu\sum_{n=0}^{\infty}q^{n} (\lambda-\lambda\mu-q^{n+1}\mu ) (1-q^{n}\mu )^{\tau} \end{array}\displaystyle \right ], &(\lambda+q)\mu>\lambda. \end{cases}\displaystyle \end{aligned}$$

### Proof

When $(\lambda+q)\mu\leq\lambda$, making use of Lemma 2.2, we get

$$\begin{aligned} \int_{0}^{\mu}t^{\tau} \bigl\vert qt-( \lambda-\lambda\mu) \bigr\vert \,{}_{0}\mathrm{d}_{q}t &= \int_{0}^{\mu}\bigl[(\lambda-\lambda \mu)t^{\tau}-qt^{\tau+1} \bigr]\,{}_{0} \mathrm{d}_{q}t \\ &=\frac{\mu^{\tau+1}(1-q)(\lambda-\lambda\mu)}{1-q^{\tau+1}}-\frac{q\mu ^{\tau+2}(1-q)}{1-q^{\tau+2}}. \end{aligned}$$

When $(\lambda+q)\mu>\lambda$, making use of Lemma 2.2 again, we get

$$\begin{aligned} & \int_{0}^{\mu}t^{\tau} \bigl\vert qt-( \lambda-\lambda\mu) \bigr\vert \,{}_{0}\mathrm {d}_{q}t \\ &\quad = \int_{0}^{\frac{\lambda-\lambda\mu}{q}} \bigl[(\lambda-\lambda\mu )t^{\tau}-qt^{\tau+1} \bigr]\,{}_{0} \mathrm{d}_{q}t + \int^{\mu}_{\frac{\lambda-\lambda\mu}{q}} \bigl[qt^{\tau+1}-(\lambda - \lambda\mu)t^{\tau} \bigr]\,{}_{0}\mathrm{d}_{q}t \\ &\quad =2 \int_{0}^{\frac{\lambda-\lambda\mu}{q}} \bigl[(\lambda-\lambda\mu )t^{\tau}-qt^{\tau+1} \bigr]\,{}_{0} \mathrm{d}_{q}t + \int^{\mu}_{0} \bigl[qt^{\tau+1}-(\lambda- \lambda\mu)t^{\tau} \bigr]\,{}_{0}\mathrm{d}_{q}t \\ &\quad =\frac{2(1-q)^{2}(\lambda-\lambda\mu)^{\tau+2}}{(1-q^{\tau +1})(1-q^{\tau+2})}+\frac{q\mu^{\tau+2}(1-q)}{1-q^{\tau+2}}-\frac{\mu ^{\tau+1}(1-q)(\lambda-\lambda\mu)}{1-q^{\tau+1}}. \end{aligned}$$

Similarly, we also get

$$\begin{aligned} & \int_{0}^{\mu}(1-t)^{\tau} \bigl\vert qt-( \lambda-\lambda\mu) \bigr\vert \,{}_{0}\mathrm {d}_{q}t \\ &\quad = \textstyle\begin{cases} (1-q)\mu\sum_{n=0}^{\infty}q^{n} (\lambda-\lambda\mu-q^{n+1}\mu ) (1-q^{n}\mu )^{\tau}, &(\lambda+q)\mu\leq\lambda, \\ \left [ \textstyle\begin{array}{l} 2(1-q)(\lambda-\lambda\mu)^{2}\sum_{n=0}^{\infty}q^{n-1} (1-q^{n} ) [1-q^{n-1}(\lambda-\lambda\mu) ]^{\tau}\\ \quad {}-(1-q)\mu\sum_{n=0}^{\infty}q^{n} (\lambda-\lambda\mu-q^{n+1}\mu ) (1-q^{n}\mu )^{\tau} \end{array}\displaystyle \right ], &(\lambda+q)\mu>\lambda. \end{cases}\displaystyle \end{aligned}$$

This completes the proof. □

The following results of Lemma 2.4, Lemma 2.5 and Lemma 2.6 are stated without proof.

### Lemma 2.4

*Let* $\lambda,\mu\in[0,1]$ *and* $\tau\in[0,\infty)$. *Then we have*

$$\begin{aligned} & \int_{0}^{1} t^{\tau} \bigl\vert qt-(1- \lambda\mu) \bigr\vert \,{}_{0}\mathrm{d}_{q}t \\ &\quad = \textstyle\begin{cases} \frac{(1-q)(1-\lambda\mu)}{1-q^{\tau+1}}-\frac{q(1-q)}{1-q^{\tau+2}}, &\lambda\mu+q\leq1, \\ \frac{2(1-q)^{2}(1-\lambda\mu)^{\tau+2}}{(1-q^{\tau+1})(1-q^{\tau +2})}+\frac{q(1-q)}{1-q^{\tau+2}}-\frac{(1-q)(1-\lambda\mu)}{1-q^{\tau+1}}, &\lambda\mu+q>1, \end{cases}\displaystyle \end{aligned}$$

*and*

$$\begin{aligned} & \int_{0}^{1} (1-t)^{\tau} \bigl\vert qt-(1-\lambda\mu) \bigr\vert \,{}_{0}\mathrm{d}_{q}t \\ &\quad = \textstyle\begin{cases} (1-q)\sum_{n=0}^{\infty}q^{n} (1-\lambda\mu-q^{n+1} ) (1-q^{n} )^{\tau}, &\lambda\mu+q\leq1, \\ \left [ \textstyle\begin{array}{l} 2(1-q)(1-\lambda\mu)^{2}\sum_{n=0}^{\infty}q^{n-1} (1-q^{n} ) [1-q^{n-1}(1-\lambda\mu) ]^{\tau}\\ \quad {}-(1-q)\sum_{n=0}^{\infty}q^{n} (1-\lambda\mu-q^{n+1} ) (1-q^{n} )^{\tau} \end{array}\displaystyle \right ], &\lambda\mu+q>1. \end{cases}\displaystyle \end{aligned}$$

### Lemma 2.5

*Let* $\lambda,\mu\in[0,1]$ *and* $\tau\in[0,\infty)$. *Then we have*

$$\begin{aligned} & \int_{0}^{\mu}t^{\tau} \bigl\vert qt-(1- \lambda\mu) \bigr\vert \,{}_{0}\mathrm{d}_{q}t \\ &\quad = \textstyle\begin{cases} \frac{\mu^{\tau+1}(1-\lambda\mu)(1-q)}{1-q^{\tau+1}}-\frac{q\mu^{\tau +2}(1-q)}{1-q^{\tau+2}}, &(\lambda+q)\mu\leq1, \\ \frac{2(1-q)^{2}(1-\lambda\mu)^{\tau+2}}{(1-q^{\tau+1})(1-q^{\tau +2})}+\frac{q\mu^{\tau+2}(1-q)}{1-q^{\tau+2}}-\frac{\mu^{\tau +1}(1-\lambda\mu)(1-q)}{1-q^{\tau+1}}, &(\lambda+q)\mu> 1, \end{cases}\displaystyle \end{aligned}$$

*and*

$$\begin{aligned} & \int_{0}^{\mu}(1-t)^{\tau} \bigl\vert qt-(1-\lambda\mu) \bigr\vert \,{}_{0}\mathrm{d}_{q}t \\ &\quad = \textstyle\begin{cases} (1-q)\mu\sum_{n=0}^{\infty}q^{n} (1-\lambda\mu-q^{n+1}\mu ) (1-q^{n}\mu )^{\tau}, &(\lambda+q)\mu\leq1, \\ \left [ \textstyle\begin{array}{l} 2(1-q)(1-\lambda\mu)^{2}\sum_{n=0}^{\infty}q^{n-1} (1-q^{n} ) [1-q^{n-1}(1-\lambda\mu) ]^{\tau}\\ \quad {}-(1-q)\mu\sum_{n=0}^{\infty}q^{n} (1-\lambda\mu-q^{n+1}\mu ) (1-q^{n}\mu )^{\tau} \end{array}\displaystyle \right ], &(\lambda+q)\mu>1. \end{cases}\displaystyle \end{aligned}$$

### Lemma 2.6

*Let* $\lambda,\mu\in[0,1]$ *and* $\theta\in[1,\infty)$. *Then we have*

$$\begin{aligned} & \int_{0}^{1} \bigl\vert qt-(1-\lambda\mu) \bigr\vert ^{\theta}\,{}_{0}\mathrm{d}_{q}t \\ &\quad = \textstyle\begin{cases} (1-q)\sum_{n=0}^{\infty}q^{n} (1-\lambda\mu-q^{n+1} )^{\theta}, &0\leq\lambda\mu\leq1-q, \\ \left [ \textstyle\begin{array}{l} (1-q)(1-\lambda\mu)^{\theta+1}\sum_{n=0}^{\infty}q^{n-1} (1-q^{n} )^{\theta}\\ \quad {}+(1-q)\sum_{n=0}^{\infty}q^{n} (q^{n+1}-1+\lambda\mu )^{\theta}\\ \quad {}-(1-q)(1-\lambda\mu)^{\theta+1}\sum_{n=0}^{\infty}q^{n-1} (q^{n}-1 )^{\theta}\end{array}\displaystyle \right ], &1-q< \lambda\mu\leq1. \end{cases}\displaystyle \end{aligned}$$

## Main results

In 2018, Alp et al. established the *q*-Hermite–Hadamard inequality in [2]. Here we give a new proof, which is more concise.

### Theorem 3.1

*Let* $f:I\rightarrow\mathbb{R}$ *be a convex function on* $[a,b]$ *with* $0< q<1$. *Then we have*

$$ f \biggl(\frac{qa+b}{1+q} \biggr)\leq\frac{1}{b-a} \int_{a}^{b}f(x)\,{}_{a}\mathrm {d}_{q}x\leq\frac{qf(a)+f(b)}{1+q}. $$

### Proof

It is obvious that $\sum_{n=0}^{\infty}(1-q)q^{n}=1$, $0< q<1$. Since Jensen’s inequality defined on convex sets for infinite sums still remains true, utilizing this fact and Definition 1.2, we have

$$\begin{aligned} f \biggl(\frac{qa+b}{1+q} \biggr) &=f \Biggl(\sum _{n=0}^{\infty}(1-q)q^{n} \bigl(q^{n}b+ \bigl(1-q^{n}\bigr)a \bigr) \Biggr) \\ &\leq\sum_{n=0}^{\infty}(1-q)q^{n}f \bigl(q^{n}b+\bigl(1-q^{n}\bigr)a \bigr) \\ &=\frac{1}{b-a} \int_{a}^{b}f(x)\,{}_{a} \mathrm{d}_{q}x. \end{aligned}$$

Using Definition 1.2 and the convexity of *f*, we get

$$\begin{aligned} \frac{1}{b-a} \int_{a}^{b}f(x)\,{}_{a} \mathrm{d}_{q}x &= \sum_{n=0}^{\infty}(1-q)q^{n}f \bigl(q^{n}b+\bigl(1-q^{n}\bigr)a \bigr) \\ &\leq\sum_{n=0}^{\infty}(1-q)q^{n} \bigl[q^{n}f(b)+\bigl(1-q^{n}\bigr)f(a) \bigr] \\ &=\frac{qf(a)+f(b)}{1+q}. \end{aligned}$$

The proof is completed. □

Using Lemma 2.1, we can obtain the following theorem.

### Theorem 3.2

*For* $0\leq a< b$ *and some fixed* $m\in(0,1]$, *let* $f: [a,\frac {b}{m} ]\rightarrow\mathbb{R}$ *be a continuous and* *q*-*differentiable function on* $(a,\frac{b}{m} )$, *and let* ${}_{a}D_{q}f$ *be integrable on* $[a,\frac{b}{m} ]$ *with* $0< q <1$. *Then the inequality*

$$\begin{aligned} & \biggl\vert \lambda \bigl[\mu f(b)+(1-\mu)f(a) \bigr]+(1-\lambda)f \bigl(\mu b+(1-\mu)a \bigr)-\frac{1}{b-a} \int_{a}^{b}f(x)\,{}_{a} \mathrm{d}_{q}x \biggr\vert \\ &\quad \leq\min \bigl\{ \mathcal{H}_{1}(\lambda,\mu,\alpha,m),\mathcal {H}_{2}(\lambda,\mu,\alpha,m) \bigr\} \end{aligned}$$

*holds for all* $\lambda,\mu\in[0,1]$ *if* $|{}_{a}D_{q}f|$ *is* $(\alpha ,m)$-*convex on* $[a,\frac{b}{m} ]$ *with* $\alpha,m\in(0,1]^{2}$, *where*

3.1 $$\begin{aligned}& \begin{aligned} \mathcal{H}_{1}(\lambda,\mu,\alpha,m) &=(b-a) \biggl\{ \bigl[\Phi_{1}(\lambda,\mu,\alpha)+\Phi_{2}( \lambda,\mu,\alpha )-\Phi_{3}(\lambda,\mu,\alpha) \bigr] \bigl\vert {}_{a}D_{q}f(b) \bigr\vert \\ &\quad {} +m \bigl[\Phi_{4}(\lambda,\mu)+\Phi_{5}(\lambda, \mu)-\Phi_{6}(\lambda,\mu )-\Phi_{1}(\lambda,\mu,\alpha) \\ &\quad {} -\Phi_{2}(\lambda,\mu,\alpha)+\Phi_{3}(\lambda, \mu,\alpha) \bigr] \biggl\vert {}_{a}D_{q}f \biggl( \frac{a}{m} \biggr) \biggr\vert \biggr\} , \end{aligned} \\& \begin{aligned} \mathcal{H}_{2}(\lambda,\mu,\alpha,m) &=(b-a) \biggl\{ \bigl[\Phi_{7}(\lambda,\mu,\alpha)+\Phi_{8}(\lambda,\mu, \alpha )-\Phi_{9}(\lambda,\mu,\alpha)\bigr] \bigl\vert {}_{a}D_{q}f(a) \bigr\vert \\ &\quad {} +m\bigl[\Phi_{4}(\lambda,\mu)+\Phi_{5}(\lambda, \mu)-\Phi_{6}(\lambda,\mu )-\Phi_{7}(\lambda,\mu,\alpha) \\ &\quad {} -\Phi_{8}(\lambda,\mu,\alpha)+\Phi_{9}(\lambda, \mu,\alpha)\bigr] \biggl\vert {}_{a}D_{q}f\biggl( \frac{b}{m}\biggr) \biggr\vert \biggr\} , \end{aligned} \\ & \begin{aligned}\Phi_{1}(\lambda,\mu,\alpha)&= \int_{0}^{\mu}t^{\alpha} \bigl\vert qt-( \lambda -\lambda\mu) \bigr\vert \,{}_{0}\mathrm{d}_{q}t \\ &= \textstyle\begin{cases} \frac{\mu^{\alpha+1}(1-q)(\lambda-\lambda\mu)}{1-q^{\alpha+1}}-\frac {q\mu^{\alpha+2}(1-q)}{1-q^{\alpha+2}}, &(\lambda+q)\mu\leq\lambda, \\ \frac{2(1-q)^{2}(\lambda-\lambda\mu)^{\alpha+2}}{(1-q^{\alpha +1})(1-q^{\alpha+2})}+\frac{q\mu^{\alpha+2}(1-q)}{1-q^{\alpha+2}}-\frac {\mu^{\alpha+1}(1-q)(\lambda-\lambda\mu)}{1-q^{\alpha+1}}, &(\lambda+q)\mu>\lambda, \end{cases}\displaystyle \end{aligned} \\ & \begin{aligned}[b] \Phi_{2}(\lambda,\mu,\alpha)&= \int_{0}^{1} t^{\alpha} \bigl\vert qt-(1- \lambda\mu ) \bigr\vert \,{}_{0}\mathrm{d}_{q}t \\ &= \textstyle\begin{cases} \frac{(1-q)(1-\lambda\mu)}{1-q^{\alpha+1}}-\frac{q(1-q)}{1-q^{\alpha+2}}, &\lambda\mu+q\leq1, \\ \frac{2(1-q)^{2}(1-\lambda\mu)^{\alpha+2}}{(1-q^{\alpha+1})(1-q^{\alpha +2})}+\frac{q(1-q)}{1-q^{\alpha+2}}-\frac{(1-q)(1-\lambda\mu )}{1-q^{\alpha+1}}, &\lambda\mu+q>1, \end{cases}\displaystyle \end{aligned} \end{aligned}$$

3.2 $$\begin{aligned}& \begin{aligned} \Phi_{3}(\lambda,\mu,\alpha)&= \int_{0}^{\mu}t^{\alpha} \bigl\vert qt-(1- \lambda\mu ) \bigr\vert \,{}_{0}\mathrm{d}_{q}t \\ &= \textstyle\begin{cases} \frac{\mu^{\alpha+1}(1-\lambda\mu)(1-q)}{1-q^{\alpha+1}}-\frac{q\mu ^{\alpha+2}(1-q)}{1-q^{\alpha+2}}, &(\lambda+q)\mu\leq1, \\ \frac{2(1-q)^{2}(1-\lambda\mu)^{\alpha+2}}{(1-q^{\alpha+1})(1-q^{\alpha +2})}+\frac{q\mu^{\alpha+2}(1-q)}{1-q^{\alpha+2}}-\frac{\mu^{\alpha +1}(1-\lambda\mu)(1-q)}{1-q^{\alpha+1}}, &(\lambda+q)\mu> 1, \end{cases}\displaystyle \end{aligned} \\ & \begin{aligned} \Phi_{4}(\lambda,\mu)&= \int_{0}^{\mu}\bigl\vert qt-(\lambda-\lambda\mu) \bigr\vert \,{}_{0}\mathrm{d}_{q}t \\ &= \textstyle\begin{cases} \lambda\mu(1-\mu)-\frac{q\mu^{2}}{1+q}, &(\lambda+q)\mu\leq\lambda, \\ \frac{2(\lambda-\lambda\mu)^{2}}{1+q}+\frac{q\mu^{2}}{1+q}- \lambda\mu (1-\mu), &(\lambda+q)\mu>\lambda, \end{cases}\displaystyle \end{aligned} \\ & \begin{aligned}[b] \Phi_{5}(\lambda,\mu)&= \int_{0}^{1} \bigl\vert qt-(1-\lambda\mu) \bigr\vert \,{}_{0}\mathrm {d}_{q}t \\ &= \textstyle\begin{cases} \frac{1}{1+q}-\lambda\mu, &\lambda\mu+q\leq1, \\ \frac{2(1-\lambda\mu)^{2}}{1+q}+\lambda\mu-\frac{1}{1+q}, &\lambda\mu+q>1, \end{cases}\displaystyle \end{aligned} \end{aligned}$$

3.3 $$\begin{aligned}& \begin{aligned} \Phi_{6}(\lambda,\mu) & = \int_{0}^{\mu}\bigl\vert qt-(1-\lambda\mu) \bigr\vert \,{}_{0}\mathrm{d}_{q}t \\ & = \textstyle\begin{cases} \mu(1-\lambda\mu)-\frac{q\mu^{2}}{1+q}, &(\lambda+q)\mu\leq1, \\ \frac{2(1-\lambda\mu)^{2}}{1+q}+\frac{q\mu^{2}}{1+q}-\mu(1-\lambda\mu), &(\lambda+q)\mu> 1, \end{cases}\displaystyle \end{aligned} \\ & \begin{aligned} &\Phi_{7}(\lambda,\mu,\alpha) \\ &\quad = \int_{0}^{\mu}(1-t)^{\alpha} \bigl\vert qt-( \lambda-\lambda\mu) \bigr\vert \,{}_{0}\mathrm {d}_{q}t \\ &\quad = \textstyle\begin{cases} (1-q)\mu\sum_{n=0}^{\infty}q^{n} (\lambda-\lambda\mu-q^{n+1}\mu ) (1-q^{n}\mu )^{\alpha}, &(\lambda+q)\mu\leq\lambda, \\ \left [ \textstyle\begin{array}{l} 2(1-q)(\lambda-\lambda\mu)^{2}\sum_{n=0}^{\infty}q^{n-1} (1-q^{n} ) [1-q^{n-1}(\lambda-\lambda\mu) ]^{\alpha}\\ \quad {}-(1-q)\mu\sum_{n=0}^{\infty}q^{n} (\lambda-\lambda\mu-q^{n+1}\mu ) (1-q^{n}\mu )^{\alpha} \end{array}\displaystyle \right ], &(\lambda+q)\mu>\lambda, \end{cases}\displaystyle \end{aligned} \\ & \begin{aligned}[b] &\Phi_{8}(\lambda,\mu,\alpha) \\ &\quad = \int_{0}^{1} (1-t)^{\alpha} \bigl\vert qt-(1-\lambda\mu) \bigr\vert \,{}_{0}\mathrm {d}_{q}t \\ &\quad = \textstyle\begin{cases} (1-q)\sum_{n=0}^{\infty}q^{n} (1-\lambda\mu-q^{n+1} ) (1-q^{n} )^{\alpha}, &\lambda\mu+q\leq1, \\ \left [ \textstyle\begin{array}{l} 2(1-q)(1-\lambda\mu)^{2}\sum_{n=0}^{\infty}q^{n-1} (1-q^{n} ) [1-q^{n-1}(1-\lambda\mu) ]^{\alpha}\\ \quad {}-(1-q)\sum_{n=0}^{\infty}q^{n} (1-\lambda\mu-q^{n+1} ) (1-q^{n} )^{\alpha} \end{array}\displaystyle \right ], &\lambda\mu+q>1, \end{cases}\displaystyle \end{aligned} \end{aligned}$$

*and*

$$ \begin{aligned} &\Phi_{9}(\lambda,\mu,\alpha) \\ &\quad = \int_{0}^{\mu}(1-t)^{\alpha} \bigl\vert qt-(1-\lambda\mu) \bigr\vert \,{}_{0}\mathrm {d}_{q}t \\ &\quad = \textstyle\begin{cases} (1-q)\mu\sum_{n=0}^{\infty}q^{n} (1-\lambda\mu-q^{n+1}\mu ) (1-q^{n}\mu )^{\alpha}, &(\lambda+q)\mu\leq1, \\ \left [ \textstyle\begin{array}{l} 2(1-q)(1-\lambda\mu)^{2}\sum_{n=0}^{\infty}q^{n-1} (1-q^{n} ) [1-q^{n-1}(1-\lambda\mu) ]^{\alpha}\\ \quad {}-(1-q)\mu\sum_{n=0}^{\infty}q^{n} (1-\lambda\mu-q^{n+1}\mu ) (1-q^{n}\mu )^{\alpha} \end{array}\displaystyle \right ], &(\lambda+q)\mu>1. \end{cases}\displaystyle \end{aligned} $$

### Proof

From Lemma 2.1, utilizing the property of the modulus and the $(\alpha,m)$-convexity of $|{}_{a}D_{q}f|$, we have

$$\begin{aligned} & \biggl\vert \lambda\bigl[\mu f(b)+(1-\mu)f(a)\bigr]+(1-\lambda)f\bigl(\mu b+(1-\mu)a\bigr)-\frac{1}{b-a} \int_{a}^{b}f(x)\,{}_{a} \mathrm{d}_{q}x \biggr\vert \\ &\quad \leq(b-a) \biggl\{ \int_{0}^{\mu} \vert qt+\lambda\mu-\lambda \vert \bigl\vert {}_{a}D_{q}f\bigl(tb+(1-t)a\bigr) \bigr\vert \,{}_{0}\mathrm{d}_{q}t \\ &\qquad {} + \int_{\mu}^{1} \vert qt+\lambda\mu-1 \vert \bigl\vert {}_{a}D_{q}f\bigl(tb+(1-t)a \bigr) \bigr\vert \,{}_{0}\mathrm{d}_{q}t \biggr\} \\ &\quad \leq(b-a) \biggl\{ \int_{0}^{\mu}\bigl\vert qt-(\lambda-\lambda\mu) \bigr\vert \biggl[t^{\alpha}\bigl\vert {}_{a}D_{q}f(b) \bigr\vert +m\bigl(1-t^{\alpha}\bigr) \biggl\vert {}_{a}D_{q}f \biggl(\frac {a}{m}\biggr) \biggr\vert \biggr]\,{}_{0} \mathrm{d}_{q}t \\ &\qquad {} + \int_{\mu}^{1} \bigl\vert qt-(1-\lambda\mu) \bigr\vert \biggl[t^{\alpha}\bigl\vert {}_{a}D_{q}f(b) \bigr\vert +m\bigl(1-t^{\alpha}\bigr) \biggl\vert {}_{a}D_{q}f \biggl(\frac{a}{m}\biggr) \biggr\vert \biggr]\,{}_{0} \mathrm{d}_{q}t \biggr\} \\ &\quad =(b-a) \biggl\{ \int_{0}^{\mu}\bigl\vert qt-(\lambda-\lambda\mu) \bigr\vert \biggl[t^{\alpha}\bigl\vert {}_{a}D_{q}f(b) \bigr\vert +m\bigl(1-t^{\alpha}\bigr) \biggl\vert {}_{a}D_{q}f \biggl(\frac {a}{m}\biggr) \biggr\vert \biggr]\,{}_{0} \mathrm{d}_{q}t \\ &\qquad {} + \int_{0}^{1} \bigl\vert qt-(1-\lambda\mu) \bigr\vert \biggl[t^{\alpha}\bigl\vert {}_{a}D_{q}f(b) \bigr\vert +m\bigl(1-t^{\alpha}\bigr) \biggl\vert {}_{a}D_{q}f \biggl(\frac{a}{m}\biggr) \biggr\vert \biggr]\,{}_{0} \mathrm{d}_{q}t \\ &\qquad {} - \int_{0}^{\mu}\bigl\vert qt-(1-\lambda\mu) \bigr\vert \biggl[t^{\alpha}\bigl\vert {}_{a}D_{q}f(b) \bigr\vert +m\bigl(1-t^{\alpha}\bigr) \biggl\vert {}_{a}D_{q}f \biggl(\frac{a}{m}\biggr) \biggr\vert \biggr]\,{}_{0} \mathrm{d}_{q}t \biggr\} \\ &\quad =(b-a) \biggl\{ \biggl[ \int_{0}^{\mu}t^{\alpha}\bigl\vert qt-( \lambda-\lambda\mu) \bigr\vert \,{}_{0}\mathrm{d}_{q}t + \int_{0}^{1} t^{\alpha}\bigl\vert qt-(1- \lambda\mu) \bigr\vert \,{}_{0}\mathrm{d}_{q}t \\ &\qquad {} - \int_{0}^{\mu}t^{\alpha}\bigl\vert qt-(1- \lambda\mu) \bigr\vert \,{}_{0}\mathrm{d}_{q}t \biggr] \bigl\vert {}_{a}D_{q}f(b) \bigr\vert \\ &\qquad {} +m \biggl[ \int_{0}^{\mu}\bigl\vert qt-(\lambda-\lambda\mu) \bigr\vert \,{}_{0}\mathrm{d}_{q}t + \int_{0}^{1} \bigl\vert qt-(1-\lambda\mu) \bigr\vert \,{}_{0}\mathrm{d}_{q}t \\ &\qquad {} - \int_{0}^{\mu}\bigl\vert qt-(1-\lambda\mu) \bigr\vert \,{}_{0}\mathrm{d}_{q}t - \int_{0}^{\mu}t^{\alpha}\bigl\vert qt-( \lambda-\lambda\mu) \bigr\vert \,{}_{0}\mathrm {d}_{q}t \\ &\qquad {} - \int_{0}^{1} t^{\alpha}\bigl\vert qt-(1- \lambda\mu) \bigr\vert \,{}_{0}\mathrm{d}_{q}t + \int_{0}^{\mu}t^{\alpha}\bigl\vert qt-(1- \lambda\mu) \bigr\vert \,{}_{0}\mathrm{d}_{q}t \biggr] \biggl\vert {}_{a}D_{q}f\biggl(\frac{a}{m}\biggr) \biggr\vert \biggr\} . \end{aligned}$$

Similarly, we get

$$\begin{aligned} & \biggl\vert \lambda\bigl[\mu f(b)+(1-\mu)f(a)\bigr]+(1-\lambda)f\bigl(\mu b+(1-\mu)a\bigr)-\frac{1}{b-a} \int_{a}^{b}f(x)\,{}_{a} \mathrm{d}_{q}x \biggr\vert \\ &\quad \leq(b-a) \biggl\{ \biggl[ \int_{0}^{\mu}(1-t)^{\alpha}\bigl\vert qt-( \lambda-\lambda \mu) \bigr\vert \,{}_{0}\mathrm{d}_{q}t + \int_{0}^{1} (1-t)^{\alpha}\bigl\vert qt-(1-\lambda\mu) \bigr\vert \,{}_{0}\mathrm{d}_{q}t \\ &\qquad {} - \int_{0}^{\mu}(1-t)^{\alpha}\bigl\vert qt-(1-\lambda\mu) \bigr\vert \,{}_{0}\mathrm {d}_{q}t \biggr] \bigl\vert {}_{a}D_{q}f(a) \bigr\vert \\ &\qquad {} +m\biggl[ \int_{0}^{\mu}\bigl\vert qt-(\lambda-\lambda\mu) \bigr\vert \,{}_{0}\mathrm{d}_{q}t + \int_{0}^{1} \bigl\vert qt-(1-\lambda\mu) \bigr\vert \,{}_{0}\mathrm{d}_{q}t \\ &\qquad {} - \int_{0}^{\mu}\bigl\vert qt-(1-\lambda\mu) \bigr\vert \,{}_{0}\mathrm{d}_{q}t - \int_{0}^{\mu}(1-t)^{\alpha}\bigl\vert qt-( \lambda-\lambda\mu) \bigr\vert \,{}_{0}\mathrm {d}_{q}t \\ &\qquad {} - \int_{0}^{1} (1-t)^{\alpha}\bigl\vert qt-(1-\lambda\mu) \bigr\vert \,{}_{0}\mathrm{d}_{q}t + \int_{0}^{\mu}(1-t)^{\alpha}\bigl\vert qt-(1-\lambda\mu) \bigr\vert \,{}_{0}\mathrm {d}_{q}t \biggr] \biggl\vert {}_{a}D_{q}f\biggl(\frac{b}{m} \biggr) \biggr\vert \biggr\} . \end{aligned}$$

Using Lemma 2.3, Lemma 2.4 and Lemma 2.5, we get the desired result. This completes the proof. □

### Corollary 3.1

*In Theorem *3.2, *putting* $\mu=\frac{1}{1+q}$, *we have*

$$\begin{aligned} & \biggl\vert \lambda\frac{qf(a)+f(b)}{1+q}+(1-\lambda)f \biggl(\frac {qa+b}{1+q} \biggr)-\frac{1}{b-a} \int_{a}^{b}f(x)\,{}_{a} \mathrm{d}_{q}x \biggr\vert \\ &\quad \leq\min \biggl\{ \mathcal{H}_{1} \biggl(\lambda, \frac{1}{1+q},\alpha,m \biggr),\mathcal{H}_{2} \biggl(\lambda, \frac{1}{1+q},\alpha,m \biggr) \biggr\} . \end{aligned}$$

### Remark 3.1

Consider Corollary 3.1.

(i) Putting $\lambda=0$, we get the midpoint-like integral inequality

$$\begin{aligned} & \biggl\vert f \biggl(\frac{qa+b}{1+q} \biggr)-\frac{1}{b-a} \int _{a}^{b}f(x)\,{}_{a} \mathrm{d}_{q}x \biggr\vert \\ &\quad \leq\min \biggl\{ \mathcal{H}_{1} \biggl(0,\frac{1}{1+q}, \alpha,m \biggr),\mathcal{H}_{2} \biggl(0,\frac{1}{1+q},\alpha,m \biggr) \biggr\} , \end{aligned}$$

where

$$\begin{aligned} &\mathcal{H}_{1} \biggl(0,\frac{1}{1+q},\alpha,m \biggr) \\ &\quad =(b-a) \biggl\{ \frac{[(1+q)^{\alpha+2}-(1+q^{\alpha +2})](1-q)^{2}}{(1+q)^{\alpha+2}(1-q^{\alpha+1})(1-q^{\alpha+2})} \bigl\vert {}_{a}D_{q}f(b) \bigr\vert \\ &\qquad {} +m \biggl[\frac{2q}{(1+q)^{3}}-\frac{[(1+q)^{\alpha+2}-(1+q^{\alpha +2})](1-q)^{2}}{(1+q)^{\alpha+2}(1-q^{\alpha+1})(1-q^{\alpha+2})} \biggr] \biggl\vert {}_{a}D_{q}f \biggl(\frac{a}{m} \biggr) \biggr\vert \biggr\} \end{aligned}$$

and

$$\begin{aligned} &\mathcal{H}_{2}\biggl(0,\frac{1}{1+q},\alpha,m\biggr) \\ &\quad = (b-a)\biggl\{ \biggl[\Phi_{7}\biggl(0,\frac{1}{1+q}, \alpha\biggr)+\Phi _{8}\biggl(0,\frac{1}{1+q},\alpha\biggr)- \Phi_{9}\biggl(0,\frac{1}{1+q},\alpha \biggr)\biggr] \bigl\vert {}_{a}D_{q}f(a) \bigr\vert \\ &\qquad {} +m\biggl[\frac{2q}{(1+q)^{3}}-\Phi_{7}\biggl(0, \frac{1}{1+q},\alpha \biggr)-\Phi_{8}\biggl(0,\frac{1}{1+q}, \alpha\biggr) \\ &\qquad {} +\Phi_{9}\biggl(0,\frac{1}{1+q},\alpha\biggr)\biggr] \biggl\vert {}_{a}D_{q}f \biggl(\frac{b}{m}\biggr) \biggr\vert \biggr\} . \end{aligned}$$

Specially, taking $\alpha=1=m$, we obtain

$$\begin{aligned} & \biggl\vert f \biggl(\frac{qa+b}{1+q} \biggr)-\frac{1}{b-a} \int _{a}^{b}f(x)\,{}_{a} \mathrm{d}_{q}x \biggr\vert \\ &\quad \leq(b-a) \biggl\{ \frac{3q}{(1+q)^{3}(1+q+q^{2})} \bigl\vert {}_{a}D_{q}f(b) \bigr\vert +\frac{-q+2q^{2}+2q^{3}}{(1+q)^{3}(1+q+q^{2})} \bigl\vert {}_{a}D_{q}f(a) \bigr\vert \biggr\} , \end{aligned}$$

which is established by Alp et al. in [2, Theorem 13].

(ii) Putting $\lambda=\frac{1}{3}$ and $\alpha=1=m$, we get the Simpson-like integral inequality

$$\begin{aligned} & \biggl\vert \frac{1}{3} \biggl[\frac{qf(a)+f(b)}{1+q}+2f \biggl( \frac {qa+b}{1+q} \biggr) \biggr]-\frac{1}{b-a} \int_{a}^{b}f(x)\,{}_{a} \mathrm{d}_{q}x \biggr\vert \\ &\quad \leq\min \biggl\{ \mathcal{H}_{1} \biggl(\frac{1}{3}, \frac{1}{1+q},1,1 \biggr),\mathcal{H}_{2} \biggl( \frac{1}{3},\frac{1}{1+q},1,1 \biggr) \biggr\} . \end{aligned}$$

Specially, if $q\rightarrow1^{-}$, then we obtain

$$ \biggl\vert \frac{1}{3} \biggl[\frac{f(a)+f(b)}{2}+2f \biggl( \frac{a+b}{2} \biggr) \biggr]-\frac{1}{b-a} \int_{a}^{b}f(x)\, \mathrm{d}x \biggr\vert \leq \frac{5(b-a)}{72} \bigl[ \bigl\vert f'(b) \bigr\vert + \bigl\vert f'(a) \bigr\vert \bigr], $$

which is established by Alomari et al. in [1, Corollary 1].

(iii) Putting $\lambda=\frac{1}{2}$ and $\alpha=1=m$, we get the averaged midpoint-trapezoid-like integral inequality

$$\begin{aligned} & \biggl\vert \frac{1}{2} \biggl[\frac{qf(a)+f(b)}{1+q}+f \biggl( \frac {qa+b}{1+q} \biggr) \biggr]-\frac{1}{b-a} \int_{a}^{b}f(x)\,{}_{a} \mathrm{d}_{q}x \biggr\vert \\ &\quad \leq\min \biggl\{ \mathcal{H}_{1} \biggl(\frac{1}{2}, \frac{1}{1+q},1,1 \biggr),\mathcal{H}_{2} \biggl( \frac{1}{2},\frac{1}{1+q},1,1 \biggr) \biggr\} . \end{aligned}$$

Specially, if $q\rightarrow1^{-}$, then we obtain

$$ \biggl\vert \frac{1}{2}\biggl[\frac{f(a)+f(b)}{2}+f\biggl( \frac{a+b}{2} \biggr)\biggr]-\frac{1}{b-a} \int_{a}^{b}f(x)\mathrm{d}x \biggr\vert \leq \frac{b-a}{16} \bigl[ \bigl\vert f'(b) \bigr\vert + \bigl\vert f'(a) \bigr\vert \bigr], $$

which is established by Xi and Qi in [30, Corollary 3.4].

(iv) Putting $\lambda=1$, we get the trapezoid-like integral inequality

$$\begin{aligned} & \biggl\vert \frac{qf(a)+f(b)}{1+q}-\frac{1}{b-a} \int_{a}^{b}f(x)\,{}_{a}\mathrm {d}_{q}x \biggr\vert \\ &\quad \leq\min \biggl\{ \mathcal{H}_{1}\biggl(1,\frac{1}{1+q}, \alpha,m \biggr),\mathcal{H}_{2}\biggl(1,\frac{1}{1+q},\alpha,m \biggr) \biggr\} , \end{aligned}$$

where

$$\begin{aligned} &\mathcal{H}_{1}\biggl(1,\frac{1}{1+q},\alpha,m\biggr) \\ &\quad = (b-a) \biggl\{ \frac{2q^{\alpha+2}(1-q)^{2}+q^{2}(1+q)^{\alpha +1}(1-q)(1-q^{\alpha})}{(1+q)^{\alpha+2}(1-q^{\alpha+1})(1-q^{\alpha +2})} \bigl\vert {}_{a}D_{q}f(b) \bigr\vert \\ &\qquad {} +m\biggl[\frac{2q^{2}}{(1+q)^{3}}-\frac{2q^{\alpha +2}(1-q)^{2}+q^{2}(1+q)^{\alpha+1}(1-q)(1-q^{\alpha})}{(1+q)^{\alpha +2}(1-q^{\alpha+1})(1-q^{\alpha+2})}\biggr] \biggl\vert {}_{a}D_{q}f \biggl(\frac {a}{m} \biggr) \biggr\vert \biggr\} \end{aligned}$$

and

$$\begin{aligned} &\mathcal{H}_{2} \biggl(1,\frac{1}{1+q},\alpha,m \biggr) \\ &\quad = (b-a)\biggl\{ \biggl[\Phi_{7} \biggl(1,\frac{1}{1+q}, \alpha \biggr)+\Phi _{8} \biggl(1,\frac{1}{1+q},\alpha \biggr)- \Phi_{9} \biggl(1,\frac{1}{1+q},\alpha \biggr) \biggr] \bigl\vert {}_{a}D_{q}f(a) \bigr\vert \\ &\qquad {} +m \biggl[\frac{2q^{2}}{(1+q)^{3}}-\Phi_{7} \biggl(1, \frac{1}{1+q},\alpha \biggr)-\Phi_{8} \biggl(1,\frac{1}{1+q}, \alpha \biggr) \\ &\qquad {} +\Phi_{9} \biggl(1,\frac{1}{1+q},\alpha \biggr) \biggr] \biggl\vert {}_{a}D_{q}f \biggl(\frac{b}{m} \biggr) \biggr\vert \biggr\} . \end{aligned}$$

Specially, taking $\alpha=1=m$, we obtain

$$\begin{aligned} & \biggl\vert \frac{qf(a)+f(b)}{1+q}-\frac{1}{b-a} \int_{a}^{b}f(x)\,{}_{a}\mathrm {d}_{q}x \biggr\vert \\ &\quad \leq(b-a) \biggl\{ \frac{q^{2}(1+4q+q^{2})}{(1+q)^{4}(1+q+q^{2})} \bigl\vert {}_{a}D_{q}f(b) \bigr\vert +\frac{q^{2}(1+3q^{2}+2q^{3})}{(1+q)^{4}(1+q+q^{2})} \bigl\vert {}_{a}D_{q}f(a) \bigr\vert \biggr\} , \end{aligned}$$

which is established by Sudsutad et al. in [26, Theorem 4.1].

If $|{}_{a}D_{q}f|^{r}$ for $r\geq1$ is $(\alpha,m)$-convex, then the following theorem can be obtained.

### Theorem 3.3

*For* $0\leq a< b$ *and some fixed* $m\in(0,1]$, *let* $f: [a,\frac {b}{m} ]\rightarrow\mathbb{R}$ *be a continuous and* *q*-*differentiable function on* $(a,\frac{b}{m} )$, *and let* ${}_{a}D_{q}f$ *be integrable on* $[a,\frac{b}{m} ]$ *with* $0< q <1$. *Then the inequality*

$$\begin{aligned} & \biggl\vert \lambda \bigl[\mu f(b)+(1-\mu)f(a) \bigr]+(1- \lambda)f \bigl(\mu b+(1-\mu)a \bigr)-\frac{1}{b-a} \int_{a}^{b}f(x)\,{}_{a} \mathrm{d}_{q}x \biggr\vert \\ &\quad \leq(b-a)\min \bigl\{ \mathcal{J}_{1}(\lambda,\mu,\alpha,m,r), \mathcal {J}_{2}(\lambda,\mu,\alpha,m,r) \bigr\} \end{aligned}$$

*holds for all* $\lambda,\mu\in[0,1]$ *if* $|{}_{a}D_{q}f|^{r}$ *for* $r\geq1$ *is* $(\alpha,m)$-*convex on* $[a,\frac{b}{m} ]$ *with* $\alpha,m\in (0,1]^{2}$, *where*

3.4 $$\begin{aligned}& \begin{aligned} &\mathcal{J}_{1}( \lambda,\mu,\alpha,m,r) \\ &\quad =\Phi_{5}^{1-\frac{1}{r}}(\lambda,\mu) \biggl[ \Phi_{2}(\lambda,\mu,\alpha ) \bigl\vert {}_{a}D_{q}f(b) \bigr\vert ^{r} +m\bigl(\Phi_{5}(\lambda,\mu)- \Phi_{2}(\lambda,\mu,\alpha)\bigr) \biggl\vert {}_{a}D_{q}f \biggl(\frac{a}{m}\biggr) \biggr\vert ^{r} \biggr]^{\frac{1}{r}} \\ &\qquad {} +(1-\lambda)\mu^{1-\frac{1}{r}} \biggl[\Upsilon_{1}(\mu, \alpha) \bigl\vert {}_{a}D_{q}f(b) \bigr\vert ^{r}+m\Upsilon_{2}(\mu,\alpha) \biggl\vert {}_{a}D_{q}f\biggl(\frac {a}{m}\biggr) \biggr\vert ^{r} \biggr]^{\frac{1}{r}}, \end{aligned} \\& \begin{aligned} &\mathcal{J}_{2}(\lambda,\mu, \alpha,m,r) \\ &\quad =\Phi_{5}^{1-\frac{1}{r}}(\lambda,\mu) \biggl[ \Phi_{8}(\lambda,\mu,\alpha ) \bigl\vert {}_{a}D_{q}f(a) \bigr\vert ^{r} +m\bigl(\Phi_{5}(\lambda,\mu)- \Phi_{8}(\lambda,\mu,\alpha)\bigr) \biggl\vert {}_{a}D_{q}f \biggl(\frac{b}{m}\biggr) \biggr\vert ^{r} \biggr]^{\frac{1}{r}} \\ &\qquad {} +(1-\lambda)\mu^{1-\frac{1}{r}} \biggl[\Upsilon_{3}(\mu, \alpha) \bigl\vert {}_{a}D_{q}f(a) \bigr\vert ^{r}+m\Upsilon_{4}(\mu,\alpha) \biggl\vert {}_{a}D_{q}f\biggl(\frac {b}{m}\biggr) \biggr\vert ^{r} \biggr]^{\frac{1}{r}}, \end{aligned} \\& \Upsilon_{1}(\mu,\alpha)= \int_{0}^{\mu}t^{\alpha} \,{}_{0} \mathrm{d}_{q}t=\frac {\mu^{\alpha+1}(1-q)}{1-q^{\alpha+1}}, \end{aligned}$$

3.5 $$\begin{aligned}& \Upsilon_{2}(\mu,\alpha)= \int_{0}^{\mu} \bigl(1-t^{\alpha} \bigr) \,{}_{0}\mathrm {d}_{q}t=\mu-\frac{\mu^{\alpha+1}(1-q)}{1-q^{\alpha+1}}, \end{aligned}$$

3.6 $$\begin{aligned}& \Upsilon_{3}(\mu,\alpha)= \int_{0}^{\mu}(1-t)^{\alpha} \,{}_{0}\mathrm {d}_{q}t=(1-q)\mu\sum _{n=0}^{\infty}q^{n} \bigl(1-q^{n}\mu \bigr)^{\alpha}, \end{aligned}$$

3.7 $$\begin{aligned}& \Upsilon_{4}(\mu,\alpha)= \int_{0}^{\mu} \bigl(1-(1-t)^{\alpha} \bigr) \,{}_{0}\mathrm{d}_{q}t=\mu-(1-q)\mu\sum _{n=0}^{\infty}q^{n} \bigl(1-q^{n}\mu \bigr)^{\alpha}, \end{aligned}$$

*and* $\Phi_{2}(\lambda,\mu,\alpha)$, $\Phi_{5}(\lambda,\mu)$, $\Phi_{8}(\lambda ,\mu,\alpha)$ *are defined by* (3.1), (3.2) *and* (3.3), *respectively*.

### Proof

Using Lemma 2.1 and the power mean inequality, we have

3.8 $$\begin{aligned} & \biggl\vert \lambda\bigl[\mu f(b)+(1-\mu)f(a)\bigr]+(1-\lambda)f\bigl(\mu b+(1-\mu)a\bigr)-\frac{1}{b-a} \int_{a}^{b}f(x)\,{}_{a} \mathrm{d}_{q}x \biggr\vert \\ &\quad \leq(b-a) \biggl\{ \biggl( \int_{0}^{1} \bigl\vert qt-(1-\lambda\mu) \bigr\vert \,{}_{0}\mathrm {d}_{q}t\biggr)^{1-\frac{1}{r}} \\ &\qquad {} \times\biggl( \int_{0}^{1} \bigl\vert qt-(1-\lambda\mu) \bigr\vert \bigl\vert {}_{a}D_{q}f \bigl(tb+(1-t)a\bigr) \bigr\vert ^{r}\,{}_{0}\mathrm{d}_{q}t \biggr)^{\frac{1}{r}} \\ &\qquad {} +(1-\lambda) \biggl( \int_{0}^{\mu}1 \,{}_{0} \mathrm{d}_{q}t\biggr)^{1-\frac{1}{r}} \biggl( \int_{0}^{\mu}\bigl\vert {}_{a}D_{q}f \bigl(tb+(1-t)a\bigr) \bigr\vert ^{r}\,{}_{0}\mathrm {d}_{q}t\biggr)^{\frac{1}{r}} \biggr\} . \end{aligned}$$

Utilizing the $(\alpha,m)$-convexity of $|{}_{a}D_{q}f|^{r}$, we get

3.9 $$\begin{aligned} & \int_{0}^{1} \bigl\vert qt-(1-\lambda\mu) \bigr\vert \bigl\vert {}_{a}D_{q}f\bigl(tb+(1-t)a \bigr) \bigr\vert ^{r}\,{}_{0}\mathrm{d}_{q}t \\ &\quad \leq \int_{0}^{1} \bigl\vert qt-(1-\lambda\mu) \bigr\vert \biggl[t^{\alpha} \bigl\vert {}_{a}D_{q}f(b) \bigr\vert ^{r}+m\bigl(1-t^{\alpha}\bigr) \biggl\vert {}_{a}D_{q}f\biggl(\frac{a}{m} \biggr) \biggr\vert ^{r} \biggr]\,{}_{0}\mathrm{d}_{q}t \\ & \quad = \biggl( \int_{0}^{1}t^{\alpha}\bigl\vert qt-(1- \lambda\mu) \bigr\vert \,{}_{0}\mathrm {d}_{q}t \biggr) \bigl\vert {}_{a}D_{q}f(b) \bigr\vert ^{r} \\ &\qquad {} +m \biggl( \int_{0}^{1} \bigl\vert qt-(1-\lambda\mu) \bigr\vert \,{}_{0}\mathrm{d}_{q}t- \int _{0}^{1}t^{\alpha} \bigl\vert qt-(1- \lambda\mu) \bigr\vert \,{}_{0}\mathrm{d}_{q}t \biggr) \biggl\vert {}_{a}D_{q}f\biggl(\frac{a}{m}\biggr) \biggr\vert ^{r} \end{aligned}$$

and

3.10 $$\begin{aligned} & \int_{0}^{\mu}\bigl\vert {}_{a}D_{q}f \bigl(tb+(1-t)a\bigr) \bigr\vert ^{r}\,{}_{0} \mathrm{d}_{q}t \\ &\quad \leq \int_{0}^{\mu}\biggl[t^{\alpha} \bigl\vert {}_{a}D_{q}f(b) \bigr\vert ^{r}+m \bigl(1-t^{\alpha }\bigr) \biggl\vert {}_{a}D_{q}f \biggl(\frac{a}{m}\biggr) \biggr\vert ^{r} \biggr] \,{}_{0}\mathrm{d}_{q}t \\ &\quad = \biggl( \int_{0}^{\mu}t^{\alpha}\,{}_{0} \mathrm{d}_{q}t \biggr) \bigl\vert {}_{a}D_{q}f(b) \bigr\vert ^{r} +m \biggl( \int_{0}^{\mu}\bigl(1-t^{\alpha}\bigr) \,{}_{0}\mathrm{d}_{q}t \biggr) \biggl\vert {}_{a}D_{q}f\biggl(\frac{a}{m}\biggr) \biggr\vert ^{r}. \end{aligned}$$

Using (3.9) and (3.10) in (3.8), we get

3.11 $$\begin{aligned} & \biggl\vert \lambda\bigl[\mu f(b)+(1-\mu)f(a)\bigr]+(1-\lambda)f\bigl(\mu b+(1-\mu)a\bigr)-\frac{1}{b-a} \int_{a}^{b}f(x)\,{}_{a} \mathrm{d}_{q}x \biggr\vert \\ &\quad \leq(b-a)\biggl\{ \biggl[ \int_{0}^{1} \bigl\vert qt-(1-\lambda\mu) \bigr\vert \,{}_{0}\mathrm {d}_{q}t\biggr]^{1-\frac{1}{r}} \\ &\qquad {} \times\biggl[\biggl( \int_{0}^{1}t^{\alpha}\bigl\vert qt-(1- \lambda\mu) \bigr\vert \,{}_{0}\mathrm{d}_{q}t\biggr) \bigl\vert {}_{a}D_{q}f(b) \bigr\vert ^{r} \\ &\qquad {} +m\biggl( \int_{0}^{1} \bigl\vert qt-(1-\lambda\mu) \bigr\vert \,{}_{0}\mathrm{d}_{q}t- \int _{0}^{1}t^{\alpha} \bigl\vert qt-(1- \lambda\mu) \bigr\vert \,{}_{0}\mathrm{d}_{q}t\biggr) \biggl\vert {}_{a}D_{q}f\biggl(\frac{a}{m}\biggr) \biggr\vert ^{r}\biggr]^{\frac{1}{r}} \\ &\qquad {} +(1-\lambda)\mu^{1-\frac{1}{r}}\biggl[\biggl( \int_{0}^{\mu}t^{\alpha }\,{}_{0} \mathrm{d}_{q}t\biggr) \bigl\vert {}_{a}D_{q}f(b) \bigr\vert ^{r} \\ &\qquad {} +m\biggl( \int_{0}^{\mu}\bigl(1-t^{\alpha}\bigr) \,{}_{0}\mathrm{d}_{q}t\biggr) \biggl\vert {}_{a}D_{q}f\biggl(\frac{a}{m}\biggr) \biggr\vert ^{r}\biggr]^{\frac{1}{r}}\biggr\} . \end{aligned}$$

Similarly, we get

3.12 $$\begin{aligned} & \int_{0}^{1} \bigl\vert qt-(1-\lambda\mu) \bigr\vert \bigl\vert {}_{a}D_{q}f\bigl(tb+(1-t)a \bigr) \bigr\vert ^{r}\,{}_{0}\mathrm{d}_{q}t \\ &\quad \leq \int_{0}^{1} \bigl\vert qt-(1-\lambda\mu) \bigr\vert \biggl[(1-t)^{\alpha} \bigl\vert {}_{a}D_{q}f(a) \bigr\vert ^{r}+m\bigl(1-(1-t)^{\alpha}\bigr) \biggl\vert {}_{a}D_{q}f\biggl(\frac {b}{m}\biggr) \biggr\vert ^{r} \biggr]\,{}_{0}\mathrm{d}_{q}t \\ &\quad = \biggl( \int_{0}^{1}(1-t)^{\alpha}\bigl\vert qt-(1- \lambda\mu) \bigr\vert \,{}_{0}\mathrm {d}_{q}t \biggr) \bigl\vert {}_{a}D_{q}f(a) \bigr\vert ^{r} \\ &\qquad {} +m \biggl( \int_{0}^{1} \bigl\vert qt-(1-\lambda\mu) \bigr\vert \,{}_{0}\mathrm{d}_{q}t- \int _{0}^{1}(1-t)^{\alpha} \bigl\vert qt-(1-\lambda\mu) \bigr\vert \,{}_{0}\mathrm{d}_{q}t \biggr) \\ &\qquad {}\times\biggl\vert {}_{a}D_{q}f\biggl(\frac{b}{m} \biggr) \biggr\vert ^{r} \end{aligned}$$

and

3.13 $$\begin{aligned} & \int_{0}^{\mu}\bigl\vert {}_{a}D_{q}f \bigl(tb+(1-t)a\bigr) \bigr\vert ^{r}\,{}_{0} \mathrm{d}_{q}t \\ &\quad \leq \int_{0}^{\mu}\biggl[(1-t)^{\alpha} \bigl\vert {}_{a}D_{q}f(a) \bigr\vert ^{r}+m \bigl(1-(1-t)^{\alpha}\bigr) \biggl\vert {}_{a}D_{q}f \biggl(\frac{b}{m}\biggr) \biggr\vert ^{r} \biggr] \,{}_{0}\mathrm{d}_{q}t \\ &\quad = \biggl( \int_{0}^{\mu}(1-t)^{\alpha}\,{}_{0} \mathrm{d}_{q}t \biggr) \bigl\vert {}_{a}D_{q}f(a) \bigr\vert ^{r} \\ &\qquad {} +m \biggl( \int_{0}^{\mu}\bigl(1-(1-t)^{\alpha}\bigr) \,{}_{0}\mathrm{d}_{q}t \biggr) \biggl\vert {}_{a}D_{q}f\biggl(\frac{b}{m}\biggr) \biggr\vert ^{r}. \end{aligned}$$

Using (3.12) and (3.13) in (3.8), we get

3.14 $$\begin{aligned} & \biggl\vert \lambda\bigl[\mu f(b)+(1-\mu)f(a)\bigr]+(1-\lambda)f\bigl(\mu b+(1-\mu)a\bigr)-\frac{1}{b-a} \int_{a}^{b}f(x)\,{}_{a} \mathrm{d}_{q}x \biggr\vert \\ & \quad \leq(b-a) \biggl\{ \biggl[ \int_{0}^{1} \bigl\vert qt-(1-\lambda\mu) \bigr\vert \,{}_{0}\mathrm {d}_{q}t\biggr]^{1-\frac{1}{r}} \\ &\qquad {} \times\biggl[\biggl( \int_{0}^{1}(1-t)^{\alpha}\bigl\vert qt-(1- \lambda\mu) \bigr\vert \,{}_{0}\mathrm{d}_{q}t\biggr) \bigl\vert {}_{a}D_{q}f(a) \bigr\vert ^{r} \\ &\qquad {} +m\biggl( \int_{0}^{1} \bigl\vert qt-(1-\lambda\mu) \bigr\vert \,{}_{0}\mathrm{d}_{q}t- \int _{0}^{1}(1-t)^{\alpha} \bigl\vert qt-(1-\lambda\mu) \bigr\vert \,{}_{0}\mathrm{d}_{q}t \biggr) \biggl\vert {}_{a}D_{q}f\biggl(\frac{b}{m} \biggr) \biggr\vert ^{r}\biggr]^{\frac{1}{r}} \\ &\qquad {} +(1-\lambda)\mu^{1-\frac{1}{r}}\biggl[\biggl( \int_{0}^{\mu}(1-t)^{\alpha }\,{}_{0} \mathrm{d}_{q}t\biggr) \bigl\vert {}_{a}D_{q}f(a) \bigr\vert ^{r} \\ &\qquad {} +m\biggl( \int_{0}^{\mu}\bigl(1-(1-t)^{\alpha}\bigr) \,{}_{0}\mathrm{d}_{q}t \biggr) \biggl\vert {}_{a}D_{q}f\biggl(\frac{b}{m}\biggr) \biggr\vert ^{r}\biggr]^{\frac{1}{r}} \biggr\} . \end{aligned}$$

From (3.11) and (3.14), utilizing (3.1), (3.2), (3.3) and Lemma 2.2, we can deduce the desired result. The proof is complete. □

If $|{}_{a}D_{q}f|^{r}$ for $r>1$ is $(\alpha,m)$-convex, then the following theorem can be obtained.

### Theorem 3.4

*For* $0\leq a< b$ *and some fixed* $m\in(0,1]$, *let* $f: [a,\frac {b}{m} ]\rightarrow\mathbb{R}$ *be a continuous and* *q*-*differentiable function on* $(a,\frac{b}{m} )$, *and let* ${}_{a}D_{q}f$ *be integrable on* $[a,\frac{b}{m} ]$ *with* $0< q <1$. *Then the inequality*

$$\begin{aligned} & \biggl\vert \lambda \bigl[\mu f(b)+(1-\mu)f(a) \bigr]+(1-\lambda)f \bigl(\mu b+(1-\mu)a \bigr)-\frac{1}{b-a} \int_{a}^{b}f(x)\,{}_{a} \mathrm{d}_{q}x \biggr\vert \\ &\quad \leq(b-a)\min \bigl\{ \mathcal{K}_{1}(\lambda,\mu,\alpha,m), \mathcal {K}_{2}(\lambda,\mu,\alpha,m) \bigr\} \end{aligned}$$

*holds for all* $\lambda,\mu\in[0,1]$ *if* $|{}_{a}D_{q}f|^{r}$ *for* $r> 1$ *with* $r^{-1}+s^{-1}=1$ *is* $(\alpha,m)$-*convex on* $[a,\frac{b}{m} ]$ *with* $\alpha,m\in(0,1]^{2}$, *where*

$$\begin{aligned}& \begin{aligned} &\mathcal{K}_{1}(\lambda,\mu,\alpha,m) \\ &\quad =\Psi_{1}^{\frac{1}{s}}(\lambda,\mu) \biggl[ \Psi_{2}(\alpha) \bigl\vert {}_{a}D_{q}f(b) \bigr\vert ^{r}+m\bigl(1-\Psi_{2}(\alpha )\bigr) \biggl\vert {}_{a}D_{q}f\biggl(\frac{a}{m}\biggr) \biggr\vert ^{r}\biggr]^{\frac{1}{r}} \\ &\qquad {} +(1-\lambda)\mu^{\frac{1}{s}} \biggl[\Upsilon_{1}(\mu, \alpha) \bigl\vert {}_{a}D_{q}f(b) \bigr\vert ^{r}+m\Upsilon_{2}(\mu ,\alpha) \biggl\vert {}_{a}D_{q}f\biggl(\frac{a}{m}\biggr) \biggr\vert ^{r}\biggr]^{\frac{1}{r}}, \end{aligned} \\& \begin{aligned} &\mathcal{K}_{2}(\lambda,\mu,\alpha,m) \\ &\quad =\Psi_{1}^{\frac{1}{s}}(\lambda,\mu) \biggl[ \Psi_{3}(\alpha) \bigl\vert {}_{a}D_{q}f(a) \bigr\vert ^{r}+m\bigl(1-\Psi_{3}(\alpha )\bigr) \biggl\vert {}_{a}D_{q}f\biggl(\frac{b}{m}\biggr) \biggr\vert ^{r}\biggr]^{\frac{1}{r}} \\ &\qquad {} +(1-\lambda)\mu^{\frac{1}{s}} \biggl[\Upsilon_{3}(\mu, \alpha) \bigl\vert {}_{a}D_{q}f(a) \bigr\vert ^{r}+m\Upsilon_{4}(\mu ,\alpha) \biggl\vert {}_{a}D_{q}f\biggl(\frac{b}{m}\biggr) \biggr\vert ^{r}\biggr]^{\frac{1}{r}}, \end{aligned} \\& \begin{aligned} \Psi_{1}(\lambda,\mu)&= \int_{0}^{1} \bigl\vert qt-(1-\lambda\mu) \bigr\vert ^{s}\,{}_{0}\mathrm{d}_{q}t \\ &= \textstyle\begin{cases} (1-q)\sum_{n=0}^{\infty}q^{n}(1-\lambda\mu-q^{n+1})^{s}, &0\leq\lambda\mu\leq1-q, \\ \left [ \textstyle\begin{array}{l} (1-q)(1-\lambda\mu)^{s+1}\sum_{n=0}^{\infty}q^{n-1}(1-q^{n})^{s}\\ \quad {}+(1-q)\sum_{n=0}^{\infty}q^{n}(q^{n+1}-1+\lambda\mu)^{s}\\ \quad {}-(1-q)(1-\lambda\mu)^{s+1}\sum_{n=0}^{\infty}q^{n-1}(q^{n}-1)^{s} \end{array}\displaystyle \right ], &1-q< \lambda\mu\leq1, \end{cases}\displaystyle \end{aligned} \\& \Psi_{2}(\alpha)= \int_{0}^{1} t^{\alpha} \,{}_{0} \mathrm{d}_{q}t=\frac {1-q}{1-q^{\alpha+1}}, \\& \Psi_{3}(\alpha)= \int_{0}^{1} (1-t)^{\alpha} \,{}_{0}\mathrm{d}_{q}t=(1-q)\sum _{n=0}^{\infty}q^{n}\bigl(1-q^{n} \bigr)^{\alpha}, \end{aligned}$$

*and* $\Upsilon_{1}(\mu,\alpha)$, $\Upsilon_{2}(\mu,\alpha)$, $\Upsilon_{3}(\mu ,\alpha)$, $\Upsilon_{4}(\mu,\alpha)$ *are defined by* (3.4), (3.5), (3.6) *and* (3.7), *respectively*.

### Proof

Using Lemma 2.1 and the Hölder inequality, we have

3.15 $$\begin{aligned} & \biggl\vert \lambda\bigl[\mu f(b)+(1-\mu)f(a)\bigr]+(1-\lambda)f\bigl(\mu b+(1-\mu)a\bigr)-\frac{1}{b-a} \int_{a}^{b}f(x)\,{}_{a} \mathrm{d}_{q}x \biggr\vert \\ &\quad \leq(b-a) \biggl\{ \biggl( \int_{0}^{1} \bigl\vert qt-(1-\lambda\mu) \bigr\vert ^{s}\,{}_{0}\mathrm{d}_{q}t \biggr)^{\frac{1}{s}} \\ &\qquad {} \times\biggl( \int_{0}^{1} \bigl\vert {}_{a}D_{q}f \bigl(tb+(1-t)a\bigr) \bigr\vert ^{r}\,{}_{0} \mathrm{d}_{q}t\biggr)^{\frac{1}{r}} \\ &\qquad {} +(1-\lambda) \biggl( \int_{0}^{\mu} 1^{s} \,{}_{0} \mathrm{d}_{q}t\biggr)^{\frac{1}{s}} \biggl( \int_{0}^{\mu} \bigl\vert {}_{a}D_{q}f \bigl(tb+(1-t)a\bigr) \bigr\vert ^{r}\,{}_{0}\mathrm {d}_{q}t\biggr)^{\frac{1}{r}} \biggr\} . \end{aligned}$$

Utilizing the $(\alpha,m)$-convexity of $|{}_{a}D_{q}f|^{r}$, we get

3.16 $$\begin{aligned} & \int_{0}^{1} \bigl\vert {}_{a}D_{q}f \bigl(tb+(1-t)a\bigr) \bigr\vert ^{r}\,{}_{0} \mathrm{d}_{q}t \\ &\quad \leq \int_{0}^{1} \biggl[t^{\alpha} \bigl\vert {}_{a}D_{q}f(b) \bigr\vert ^{r}+m \bigl(1-t^{\alpha }\bigr) \biggl\vert {}_{a}D_{q}f \biggl(\frac{a}{m}\biggr) \biggr\vert ^{r} \biggr] \,{}_{0}\mathrm{d}_{q}t \\ &\quad = \biggl( \int_{0}^{1} t^{\alpha} \,{}_{0} \mathrm{d}_{q}t \biggr) \bigl\vert {}_{a}D_{q}f(b) \bigr\vert ^{r}+m \biggl( \int_{0}^{1} \bigl(1-t^{\alpha}\bigr) \,{}_{0}\mathrm {d}_{q}t \biggr) \biggl\vert {}_{a}D_{q}f\biggl(\frac{a}{m}\biggr) \biggr\vert ^{r} \end{aligned}$$

and

3.17 $$\begin{aligned} & \int_{0}^{\mu} \bigl\vert {}_{a}D_{q}f \bigl(tb+(1-t)a\bigr) \bigr\vert ^{r}\,{}_{0}\mathrm {d}_{q}t \\ &\quad \leq \int_{0}^{\mu} \biggl[t^{\alpha} \bigl\vert {}_{a}D_{q}f(b) \bigr\vert ^{r}+m \bigl(1-t^{\alpha}\bigr) \biggl\vert {}_{a}D_{q}f \biggl(\frac{a}{m}\biggr) \biggr\vert ^{r} \biggr] \,{}_{0}\mathrm{d}_{q}t \\ &\quad = \biggl( \int_{0}^{\mu}t^{\alpha} \,{}_{0} \mathrm{d}_{q}t \biggr) \bigl\vert {}_{a}D_{q}f(b) \bigr\vert ^{r}+m \biggl( \int_{0}^{\mu}\bigl(1-t^{\alpha} \bigr) \,{}_{0}\mathrm{d}_{q}t \biggr) \biggl\vert {}_{a}D_{q}f\biggl(\frac{a}{m}\biggr) \biggr\vert ^{r}. \end{aligned}$$

Using (3.16) and (3.17) in (3.15), we get

3.18 $$\begin{aligned} & \biggl\vert \lambda\bigl[\mu f(b)+(1-\mu)f(a)\bigr]+(1-\lambda)f\bigl(\mu b+(1-\mu)a\bigr)-\frac{1}{b-a} \int_{a}^{b}f(x)\,{}_{a} \mathrm{d}_{q}x \biggr\vert \\ &\quad \leq(b-a) \biggl\{ \biggl( \int_{0}^{1} \bigl\vert qt-(1-\lambda\mu) \bigr\vert ^{s}\,{}_{0}\mathrm{d}_{q}t \biggr)^{\frac{1}{s}} \\ &\qquad {} \times\biggl[\biggl( \int_{0}^{1} t^{\alpha} \,{}_{0} \mathrm{d}_{q}t\biggr) \bigl\vert {}_{a}D_{q}f(b) \bigr\vert ^{r}+m\biggl( \int_{0}^{1} \bigl(1-t^{\alpha}\bigr) \,{}_{0}\mathrm {d}_{q}t\biggr) \biggl\vert {}_{a}D_{q}f\biggl(\frac{a}{m}\biggr) \biggr\vert ^{r}\biggr]^{\frac {1}{r}} \\ &\qquad {} +(1-\lambda)\mu^{\frac{1}{s}} \biggl[\biggl( \int_{0}^{\mu}t^{\alpha} \,{}_{0} \mathrm{d}_{q}t\biggr) \bigl\vert {}_{a}D_{q}f(b) \bigr\vert ^{r} \\ &\qquad {} +m\biggl( \int_{0}^{\mu}\bigl(1-t^{\alpha}\bigr) \,{}_{0}\mathrm{d}_{q}t\biggr) \biggl\vert {}_{a}D_{q}f\biggl(\frac{a}{m}\biggr) \biggr\vert ^{r}\biggr]^{\frac{1}{r}} \biggr\} . \end{aligned}$$

Similarly, we get

3.19 $$\begin{aligned} & \int_{0}^{1} \bigl\vert {}_{a}D_{q}f \bigl(tb+(1-t)a\bigr) \bigr\vert ^{r}\,{}_{0} \mathrm{d}_{q}t \\ &\quad \leq \int_{0}^{1} \biggl[(1-t)^{\alpha} \bigl\vert {}_{a}D_{q}f(a) \bigr\vert ^{r}+m \bigl(1-(1-t)^{\alpha}\bigr) \biggl\vert {}_{a}D_{q}f \biggl(\frac{b}{m}\biggr) \biggr\vert ^{r} \biggr] \,{}_{0}\mathrm{d}_{q}t \\ &\quad = \biggl( \int_{0}^{1} (1-t)^{\alpha} \,{}_{0}\mathrm{d}_{q}t \biggr) \bigl\vert {}_{a}D_{q}f(a) \bigr\vert ^{r} \\ &\qquad {} +m \biggl( \int_{0}^{1} \bigl(1-(1-t)^{\alpha}\bigr) \,{}_{0}\mathrm{d}_{q}t \biggr) \biggl\vert {}_{a}D_{q}f\biggl(\frac{b}{m}\biggr) \biggr\vert ^{r} \end{aligned}$$

and

3.20 $$\begin{aligned} & \int_{0}^{\mu} \bigl\vert {}_{a}D_{q}f \bigl(tb+(1-t)a\bigr) \bigr\vert ^{r}\,{}_{0}\mathrm {d}_{q}t \\ &\quad \leq \int_{0}^{\mu} \biggl[(1-t)^{\alpha} \bigl\vert {}_{a}D_{q}f(a) \bigr\vert ^{r}+m \bigl(1-(1-t)^{\alpha}\bigr) \biggl\vert {}_{a}D_{q}f \biggl(\frac{b}{m}\biggr) \biggr\vert ^{r} \biggr] \,{}_{0}\mathrm{d}_{q}t \\ &\quad = \biggl( \int_{0}^{\mu}(1-t)^{\alpha} \,{}_{0}\mathrm{d}_{q}t \biggr) \bigl\vert {}_{a}D_{q}f(a) \bigr\vert ^{r} \\ &\qquad {} +m \biggl( \int_{0}^{\mu}\bigl(1-(1-t)^{\alpha}\bigr) \,{}_{0}\mathrm{d}_{q}t \biggr) \biggl\vert {}_{a}D_{q}f\biggl(\frac{b}{m}\biggr) \biggr\vert ^{r}. \end{aligned}$$

Using (3.19) and (3.20) in (3.15), we get

3.21 $$\begin{aligned} & \biggl\vert \lambda\bigl[\mu f(b)+(1-\mu)f(a)\bigr]+(1-\lambda)f\bigl(\mu b+(1-\mu)a\bigr)-\frac{1}{b-a} \int_{a}^{b}f(x)\,{}_{a} \mathrm{d}_{q}x \biggr\vert \\ &\quad \leq(b-a) \biggl\{ \biggl( \int_{0}^{1} \bigl\vert qt-(1-\lambda\mu) \bigr\vert ^{s}\,{}_{0}\mathrm{d}_{q}t \biggr)^{\frac{1}{s}} \\ &\qquad {} \times\biggl[\biggl( \int_{0}^{1} (1-t)^{\alpha} \,{}_{0}\mathrm{d}_{q}t\biggr) \bigl\vert {}_{a}D_{q}f(a) \bigr\vert ^{r} \\ &\qquad {} +m\biggl( \int_{0}^{1} \bigl(1-(1-t)^{\alpha}\bigr) \,{}_{0}\mathrm{d}_{q}t\biggr) \biggl\vert {}_{a}D_{q}f\biggl(\frac{b}{m}\biggr) \biggr\vert ^{r}\biggr]^{\frac{1}{r}} \\ &\qquad {} +(1-\lambda)\mu^{\frac{1}{s}} \biggl[\biggl( \int_{0}^{\mu}(1-t)^{\alpha} \,{}_{0}\mathrm{d}_{q}t\biggr) \bigl\vert {}_{a}D_{q}f(a) \bigr\vert ^{r} \\ &\qquad {} +m\biggl( \int_{0}^{\mu}\bigl(1-(1-t)^{\alpha}\bigr) \,{}_{0}\mathrm{d}_{q}t \biggr) \biggl\vert {}_{a}D_{q}f\biggl(\frac{b}{m}\biggr) \biggr\vert ^{r}\biggr]^{\frac{1}{r}} \biggr\} . \end{aligned}$$

From (3.18) and (3.21), utilizing (3.4), (3.5), (3.6), (3.7), Lemma 2.2 and Lemma 2.6, we can deduce the desired result. The proof is completed. □

### Remark 3.2

For $\mu=\frac{1}{1+q}$, if we put $\lambda=0$, $\lambda=\frac{1}{3}$, $\lambda=\frac{1}{2}$ and $\lambda=1$ in Theorem 3.3 and Theorem 3.4, then we can get the midpoint-like integral inequality, the Simpson-like integral inequality, the averaged midpoint-trapezoid-like integral inequality and the trapezoid-like integral inequality, respectively.

Next we establish the *q*-integral inequalities involving the product of two $(\alpha,m)$-convex functions.

### Theorem 3.5

*For* $0\leq a< b$ *and some fixed* $m\in(0,1]$, *let* $f,g: [a,\frac {b}{m} ]\rightarrow\mathbb{R}$ *be continuous and nonnegative functions*. *Then the inequality*

$$ \frac{1}{b-a} \int_{a}^{b} f(x)g(x)\,{}_{a} \mathrm{d}_{q}x\leq\min \bigl\{ \mathcal {L}_{1}( \alpha_{1},\alpha_{2},m),\mathcal{L}_{2}( \alpha_{1},\alpha_{2},m) \bigr\} $$

*holds if* *f* *and* *g* *are* $(\alpha_{1},m)$-*convex and* $(\alpha _{2},m)$-*convex on* $[a,\frac{b}{m} ]$ *with* $\alpha_{1},\alpha_{2}\in (0,1]^{2}$, *respectively*, *where*

$$\begin{aligned} &\mathcal{L}_{1}(\alpha_{1},\alpha_{2},m) \\ &\quad = \biggl[\frac{1-q}{1-q^{\alpha_{1}+\alpha_{2}+1}}-\frac{1-q}{1-q^{\alpha _{1}+1}}-\frac{1-q}{1-q^{\alpha_{2}+1}}+1 \biggr]m^{2}f \biggl(\frac{a}{m} \biggr)g \biggl( \frac{a}{m} \biggr) \\ &\qquad {} +\frac{1-q}{1-q^{\alpha_{1}+\alpha_{2}+1}}f(b)g(b)+ \biggl[\frac {1-q}{1-q^{\alpha_{2}+1}}- \frac{1-q}{1-q^{\alpha_{1}+\alpha_{2}+1}} \biggr]mf \biggl(\frac{a}{m} \biggr)g(b) \\ &\qquad {} + \biggl[\frac{1-q}{1-q^{\alpha_{1}+1}}-\frac{1-q}{1-q^{\alpha_{1}+\alpha _{2}+1}} \biggr]mf(b)g \biggl( \frac{a}{m} \biggr), \\ &\mathcal{L}_{2}(\alpha_{1},\alpha_{2},m) \\ &\quad = \bigl[\Theta(\alpha_{1},\alpha_{2})-\Theta( \alpha_{1})-\Theta(\alpha _{2})+1 \bigr]m^{2}f \biggl(\frac{b}{m} \biggr)g \biggl(\frac{b}{m} \biggr) \\ &\qquad {} +\Theta(\alpha_{1},\alpha_{2})f(a)g(a)+ \bigl[ \Theta(\alpha_{1})-\Theta(\alpha _{1},\alpha_{2}) \bigr]mf(a)g \biggl(\frac{b}{m} \biggr) \\ &\qquad {} + \bigl[\Theta(\alpha_{2})-\Theta(\alpha_{1}, \alpha_{2}) \bigr]mf \biggl(\frac {b}{m} \biggr)g(a), \\ &\Theta(\alpha_{1},\alpha_{2})= \int_{0}^{1} (1-t)^{\alpha_{1}+\alpha_{2}} \,{}_{0}\mathrm{d}_{q}t=(1-q)\sum _{n=0}^{\infty}q^{n} \bigl(1-q^{n} \bigr)^{\alpha _{1}+\alpha_{2}}, \end{aligned}$$

*and*

$$ \Theta(\alpha_{i})= \int_{0}^{1} (1-t)^{\alpha_{i}} \,{}_{0}\mathrm{d}_{q}t=(1-q)\sum _{n=0}^{\infty}q^{n} \bigl(1-q^{n} \bigr)^{\alpha_{i}},\quad i=1,2. $$

### Proof

Using the $(\alpha_{1},m)$-convexity of *f* and the $(\alpha _{2},m)$-convexity of *g*, respectively, for all $t\in[0,1]$, we have

3.22 $$ f \bigl(tb+(1-t)a \bigr)\leq t^{\alpha_{1}}f(b)+m \bigl(1-t^{\alpha_{1}}\bigr)f \biggl(\frac {a}{m} \biggr) $$

and

3.23 $$ g \bigl(tb+(1-t)a \bigr)\leq t^{\alpha_{2}}g(b)+m \bigl(1-t^{\alpha_{2}}\bigr)g \biggl(\frac {a}{m} \biggr). $$

Multiplying (3.22) with (3.23), we get

3.24 $$\begin{aligned} &f \bigl(tb+(1-t)a \bigr)g \bigl(tb+(1-t)a \bigr) \\ &\quad \leq t^{\alpha_{1}+\alpha_{2}}f(b)g(b)+\bigl(1-t^{\alpha_{1}}\bigr) \bigl(1-t^{\alpha _{2}}\bigr)m^{2}f \biggl(\frac{a}{m} \biggr)g \biggl(\frac{a}{m} \biggr) \\ &\qquad {} +t^{\alpha_{2}}\bigl(1-t^{\alpha_{1}}\bigr)mf \biggl( \frac{a}{m} \biggr)g(b)+t^{\alpha _{1}}\bigl(1-t^{\alpha_{2}} \bigr)mf(b)g \biggl(\frac{a}{m} \biggr). \end{aligned}$$

Taking the *q*-integral for (3.24) with respect to *t* on $(0,1)$ and using Lemma 2.2, we obtain

3.25 $$\begin{aligned} & \int_{0}^{1} f \bigl(tb+(1-t)a \bigr)g \bigl(tb+(1-t)a \bigr)\,{}_{0}\mathrm{d}_{q}t \\ &\quad \leq \biggl[\frac{1-q}{1-q^{\alpha_{1}+\alpha_{2}+1}}-\frac{1-q}{1-q^{\alpha _{1}+1}}-\frac{1-q}{1-q^{\alpha_{2}+1}}+1 \biggr]m^{2}f \biggl(\frac{a}{m} \biggr)g \biggl( \frac{a}{m} \biggr) \\ &\qquad {} +\frac{1-q}{1-q^{\alpha_{1}+\alpha_{2}+1}}f(b)g(b)+ \biggl[\frac {1-q}{1-q^{\alpha_{2}+1}}- \frac{1-q}{1-q^{\alpha_{1}+\alpha_{2}+1}} \biggr]mf \biggl(\frac{a}{m} \biggr)g(b) \\ &\qquad {} + \biggl[\frac{1-q}{1-q^{\alpha_{1}+1}}-\frac{1-q}{1-q^{\alpha_{1}+\alpha _{2}+1}} \biggr]mf(b)g \biggl( \frac{a}{m} \biggr). \end{aligned}$$

Similarly, we get

3.26 $$\begin{aligned} & \int_{0}^{1} f \bigl(tb+(1-t)a \bigr)g \bigl(tb+(1-t)a \bigr)\,{}_{0}\mathrm{d}_{q}t \\ &\quad \leq \biggl( \int_{0}^{1}(1-t)^{\alpha_{1}+\alpha_{2}}\,{}_{0} \mathrm{d}_{q}t- \int _{0}^{1}(1-t)^{\alpha_{1}}\,{}_{0} \mathrm{d}_{q}t \\ &\qquad {} - \int_{0}^{1}(1-t)^{\alpha_{2}}\,{}_{0} \mathrm{d}_{q}t+1 \biggr)m^{2}f \biggl(\frac {b}{m} \biggr)g \biggl(\frac{b}{m} \biggr) \\ &\qquad {} + \biggl( \int_{0}^{1}(1-t)^{\alpha_{1}+\alpha_{2}}\,{}_{0} \mathrm{d}_{q}t \biggr)f(a)g(a) \\ &\qquad {} + \biggl( \int_{0}^{1}(1-t)^{\alpha_{1}}\,{}_{0} \mathrm{d}_{q}t- \int_{0}^{1}(1-t)^{\alpha _{1}+\alpha_{2}}\,{}_{0} \mathrm{d}_{q}t \biggr)mf(a)g \biggl(\frac{b}{m} \biggr) \\ &\qquad {} + \biggl( \int_{0}^{1}(1-t)^{\alpha_{2}}\,{}_{0} \mathrm{d}_{q}t- \int _{0}^{1}(1-t)^{\alpha_{1}+\alpha_{2}}\,{}_{0} \mathrm{d}_{q}t \biggr)mf \biggl(\frac {b}{m} \biggr)g(a). \end{aligned}$$

A simple calculation shows that

3.27 $$ \int_{0}^{1} f \bigl(tb+(1-t)a \bigr)g \bigl(tb+(1-t)a \bigr)\,{}_{0}\mathrm{d}_{q}t = \frac{1}{b-a} \int_{a}^{b} f(x)g(x)\,{}_{a} \mathrm{d}_{q}x. $$

From (3.25), (3.26) and (3.27), we obtain the desired result. This ends the proof. □

### Corollary 3.2

*In Theorem *3.5, *choosing* $\alpha_{1}=\alpha_{2}=\alpha$, *we obtain*

$$ \frac{1}{b-a} \int_{a}^{b} f(x)g(x)\,{}_{a} \mathrm{d}_{q}x\leq\min \bigl\{ \mathcal {T}_{1}(\alpha,m), \mathcal{T}_{2}(\alpha,m) \bigr\} , $$

*where*

$$\begin{aligned} \mathcal{T}_{1}(\alpha,m) &= \frac{1-q}{1-q^{2\alpha+1}}f(b)g(b)+ \biggl[ \frac{1-q}{1-q^{2\alpha +1}}-\frac{2(1-q)}{1-q^{\alpha+1}}+1 \biggr]m^{2}f \biggl( \frac{a}{m} \biggr)g \biggl(\frac{a}{m} \biggr) \\ &\quad {} +\frac{q^{\alpha+1}(1-q)(1-q^{\alpha})}{(1-q^{\alpha+1})(1-q^{2\alpha +1})}m \biggl[f \biggl(\frac{a}{m} \biggr)g(b)+f(b)g \biggl(\frac{a}{m} \biggr) \biggr] \end{aligned}$$

*and*

$$\begin{aligned} \mathcal{T}_{2}(\alpha,m) &= \Biggl[(1-q)\sum _{n=0}^{\infty}q^{n} \bigl(1-q^{n} \bigr)^{2\alpha}-2(1-q)\sum_{n=0}^{\infty}q^{n} \bigl(1-q^{n} \bigr)^{\alpha}+1 \Biggr]m^{2}f \biggl( \frac {b}{m} \biggr)g \biggl(\frac{b}{m} \biggr) \\ &\quad {} +(1-q)\sum_{n=0}^{\infty}q^{n} \bigl(1-q^{n} \bigr)^{2\alpha}f(a)g(a) \\ &\quad {} + \Biggl[(1-q)\sum_{n=0}^{\infty}q^{n} \bigl(1-q^{n} \bigr)^{\alpha}-(1-q)\sum _{n=0}^{\infty}q^{n} \bigl(1-q^{n} \bigr)^{2\alpha} \Biggr] \\ &\quad {} \times \biggl[mf(a)g \biggl(\frac{b}{m} \biggr)+mf \biggl( \frac{b}{m} \biggr)g(a) \biggr]. \end{aligned}$$

*Further*, *taking* $\alpha=1=m$, *we get*

$$\begin{aligned} &\frac{1}{b-a} \int_{a}^{b} f(x)g(x)\,{}_{a} \mathrm{d}_{q}x \\ &\quad \leq \frac{1}{1+q+q^{2}}f(b)g(b)+\frac{q(1+q^{2})}{(1+q)(1+q+q^{2})}f(a)g(a) \\ &\qquad {} +\frac{q^{2}}{(1+q)(1+q+q^{2})} \bigl[f(a)g(b)+f(b)g(a) \bigr], \end{aligned}$$

*which is established by Sudsutad et al*. *in* [26, *Theorem *4.3].

## Conclusions

In the present research, based on a new quantum integral identity with multiple parameters, we have developed some quantum error estimations of different type inequalities through $(\alpha,m)$-convexity, such as the midpoint-like inequalities, the Simpson-like inequalities, the averaged midpoint-trapezoid-like inequalities and the trapezoid-like inequalities. The inequalities derived in this work are very helpful in error estimations involved in various approximation processes. We expect that the ideas of this article will facilitate further study concerning quantum integral inequalities.
